# Reciprocal escalation of violent extremism: Experimental and longitudinal evidence from Denmark

**DOI:** 10.1093/pnasnexus/pgaf338

**Published:** 2025-10-22

**Authors:** Milan Obaidi, Robin Bergh, John F Dovidio

**Affiliations:** Department of Psychology, University of Copenhagen, Øster Farimagsgade 2A, 1353 Copenhagen, Denmark; Department of Psychology, Uppsala University, 751 05 Uppsala, Sweden; Department of Psychology, Yale University, New Haven, CT 06510, USA

**Keywords:** intergroup conflict, Islamist-inspired and right-wing extremism, reciprocal extremism, threat perceptions, violent behavioral intentions

## Abstract

Recent surges in intergroup conflict across racial, religious, and national lines highlight how mutual perceptions of threat can lead to escalating cycles of hostility. These cycles are believed to be driven by interconnected psychological, sociopolitical, and cultural mechanisms, with each group responding aggressively to perceived hostility from the other. Yet, systematic exploration of the psychological mechanisms behind reciprocally escalating violence and negativity remains limited. This research uniquely integrates experimental and longitudinal methodologies to provide novel, causal insights into these dynamics by being the first to demonstrate reciprocal violent intentions over time between majority and minority groups. This research uniquely integrates experimental and longitudinal methodologies to provide novel, causal insights into these dynamics. Using intergroup tensions in Denmark as a case in point, we investigated escalating reciprocal violent intentions between members of a majority group and a marginalized minority. In wave 1, experiments 1A and 2A showed that both groups expressed stronger endorsement of violent extremism against the other group when they perceived hostility from that group. In wave 2, experiments 1B and 2B provided evidence of a stronger effect and a reciprocal dynamic, demonstrating how intergroup conflict can escalate in tandem toward mutual hostility.

Significance Statement Using a rigorous and novel mixed longitudinal and experimental design across two waves and four experimental manipulations, this research addresses a critical gap in empirical research exploring an urgent and timely issue: the reciprocal escalation of violent extremism. Unlike prior work, which has primarily focused on the initial stage of conflict escalation, this study demonstrates a vicious cycle of gradual escalation of conflict beyond the initial act of hostility. With violent extremism posing a growing threat globally, these findings provide insight into the psychological mechanisms underlying mutual violence escalation, which is crucial in breaking the cycles of retaliation and fostering more peaceful intergroup relations.

## Introduction

Group conflicts are often described as a vicious cycle in which each party responds to hostility from the other group with more hostility of their own (1–3). Scholars from different domains of social sciences, including psychology (3, 4), political science (1, 5), sociology (6), history (7), theology (8), and economics (2), have written about this notion. Patterns in real-world intergroup relations also suggest that reciprocal hostility can increase over time. For instance, hate crimes and hostility in Western countries toward Muslim people spike after Islamist-inspired terrorist attacks against Western nations (9, 10). Similarly, Western military interventions and foreign occupation are considered major drivers of Islamist-inspired violent extremism (11, 12).

The dynamics of group conflict, in which actions taken by one group provoke a response from the other, can lead to a cycle of escalation, with members of each group perceiving that they are reacting defensively. Despite the vast theoretical interest in group conflicts and their societal relevance, the causal dynamics behind such escalations are not fully elucidated. The current study aims to systematically test this cycle of violent conflict through an innovative combination of experimental and longitudinal designs. By leveraging psychological theories, sociological insights, and research on intergroup relations, the present research aims to provide a comprehensive understanding of the dynamics that drive the escalation of violent extremism.

### The missing causal evidence behind intergroup conflict escalation

Experimental and longitudinal studies of escalating group conflicts are rare (13, 14). Several studies have nonexperimentally explored the escalation of intergroup conflict in the context of various ethnic and religious conflicts (2, 6), for example, in comparative case studies (15), but these trace the potential source of conflict escalation after the fact (16). While such studies illustrate the plausibility of a vicious cycle, they fail to provide direct causal evidence for it and cannot rule out spurious associations and alternative explanations.

There is evidence of escalating negative relations between individuals and groups in a range of psychological phenomena. For instance, in research using the Prisoner's Dilemma and other social dilemma paradigms, reciprocal, “tit-for-tat” processes often lead to mutually competitive responses between individuals, leading to the least favorable joint outcomes for the two parties (17). Moreover, this process of escalating competition and conflict occurs even more strongly in exchanges between groups than between individuals, in part because intergroup encounters tend to arouse fear and suspicion more readily than do interpersonal interactions (18).

Research on collective action further suggests how negative intergroup experiences can increase intergroup conflict over time (19). Negative experiences in social contact with the majority-group members often increase minority-group members’ motivation to engage in collective action, in part because such experiences make perceived discrimination more salient (20). At the same time, majority-group members who experience negative contact with minority-group members show greater resistance to social changes benefitting the minority group (21). Moreover, collective action by minority-group members frequently mobilizes reactionary collective movements among majority-group members (22).

While prior research has suggested that reciprocal dynamics can escalate intergroup conflict, it has largely relied on artificial group settings, one-shot interactions, and low-stakes scenarios. The current project advances the field by testing these dynamics in real-world intergroup contexts, using a multi-wave, longitudinal design that captures tit-for-tat escalation over time.

Although experiments on unilateral escalation of conflict have examined potential triggers that can produce reciprocal escalation, such as the extent to which one group's negativity or perceptions of another group's beliefs (i.e. meta-perceptions (23)) lead that group to display greater intergroup hostility (24, 25), they do not directly investigate the iterative escalation of conflict. The present research focused on the dynamics of the escalation sequence (26), which involves a series of reciprocal steps.

In the present research, we investigated the role of intergroup threat in the reciprocal escalation of intergroup conflict, experimentally and longitudinally. Experiencing threats from another group arouses a range of biases toward that group (27). Although that threat is often in the form of direct danger to the well-being of one's group (realistic threat, for example, in terms of the threat of physical harm or the loss of valued resources), it also occurs symbolically through perceptions that the standards, beliefs, or culture of another group adversely affect core elements of the ingroup's social identity (28). The impact of symbolic threat on intergroup bias and conflict is often as potent as realistic threats (27, 28), and symbolic and realistic threats tend to be highly interrelated (29). What distinguishes the present research is that it considers the impact of threat within the escalation sequence that produces, iteratively, endorsement of intergroup violence separately among majority- and minority-group members.

Although cycles of reciprocal intergroup escalation can emerge between a wide range of conflicting social, racial, religious, or national groups, the current research focuses on Muslim and non-Muslim populations in the West, as intergroup relations between these groups have become increasingly strained in recent years.

In recent years, the Muslim versus non-Muslim rhetoric has become particularly pronounced in Denmark (30). Muslims in Denmark experience significant levels of discrimination compared to other EU countries (31). Similar patterns appear across many Western contexts: Muslims are frequently portrayed as violent (32, 33) and as culturally incompatible with Western norms and values (34, 35)—perceptions linked to heightened threat and prejudice (36–39). Such dynamics can escalate intergroup tensions. A prominent example concerns the Danish and French Muhammad cartoons, which different sides have interpreted as either free-speech defences or group-based disparagement (40); subsequent retaliatory reaction has been framed as a response to prior provocation.

We do not examine these geopolitical dynamics directly, but rather we investigate the broader psychological and intergroup mechanisms of conflict escalation—specifically, how perceived threat and hostility from one group can fuel reciprocal hostility from the other. Denmark, the first country to publish the Muhammad cartoons, provides a particularly salient national context in which tensions between Muslims and non-Muslims are well documented. Moving beyond theoretical and anecdotal accounts, the current research presents an empirical test of reciprocal conflict escalation between these groups within a Western national context.

## The current studies

To capture the dynamic process of iterative, escalating intergroup hostility and violent intentions, we employed an elaborate and novel design that combined experimental and longitudinal features within a Muslim Dane sample and a non-Muslim Dane sample (henceforth referred to as non-Muslims) in Denmark. The manipulations were connected across the two samples, so we used reactions expressed in one sample as input for manipulations in the other to capture the dynamics of escalating hostility over time.

We began by randomly selecting half of our non-Muslim sample of participants and presenting them with research findings indicating that some Muslim people in Scandinavia endorse violent extremism against the non-Muslim majority (experimental condition, experiment 1A). The information represented a description of our actual research findings (i.e. the information had a kernel of truth) but was embellished with populist anti-Muslim rhetoric, modeled after what far-right parties in Denmark have used. The other half of the non-Muslim majority sample received information unrelated to violence or relations with Muslims (control condition).

After learning that the non-Muslim participants in the experimental condition reacted with violent intentions of their own, we took that information and presented it to half of our Muslim sample (experimental condition, experiment 2A), a minority group in Denmark. In other words, consistent with the actual findings, we told them that non-Muslim participants had become more hostile toward Muslim people. The other half of the Muslim sample received irrelevant information (control condition).

Next, we went back to participants in the experimental condition within the non-Muslim sample and presented the initial results from the Muslim sample: Upon learning about the reactions from the non-Muslim respondents, Muslim participants expressed greater endorsement of violence (experiment 1B). Again, we examined the reactions among non-Muslims upon receiving this information. Finally, we presented the respondents in the Muslim experimental condition with the last piece of information: The non-Muslim respondents had expressed even further violent intentions (experiment 2B).

Expressed succinctly, we went back and forth between the non-Muslim and Muslim respondents with reports of violent intentions in the other groups. Crucially, the study involved no hypothetical scenarios or exposure to bogus information, as commonly used in social psychology experiments (41). Instead, we documented a realistic escalation of violent intentions among Muslim and non-Muslim people in Scandinavia based on learning about actual reactions in the other groups. To assess violent behavioral intentions, we employed a measure that has been previously validated among individuals involved in Islamist-inspired violent extremism (42).

Due to conceptual and methodological linkages between the separate experiments, we first describe the methods of all experiments and then present the results from all of them, as they speak to a vicious cycle of intergroup hostility. In experiment 1A, we hypothesized, based on research indicating that threat increases intergroup bias (27, 28) and violent extremism (43) that non-Muslim participants would show higher levels of threat and display greater violent intentions toward Muslims upon exposure to information indicating that Muslims endorsed violence against them. Specifically, this reaction was anticipated when participants were informed that the non-Muslims viewed their religion, norms, and values as backwards, compared with a control condition where they encountered material unrelated to Muslims. In experiment 2A, we hypothesized that the negative sentiments directed at Muslims from non-Muslims, as observed in experiment 1A, would lead to an increased endorsement of violent intentions among Muslims. This would further demonstrate that forms of outgroup hostility, like Islamophobia and Muslim extremism, reciprocally reinforce one another.

**Fig. 1.**
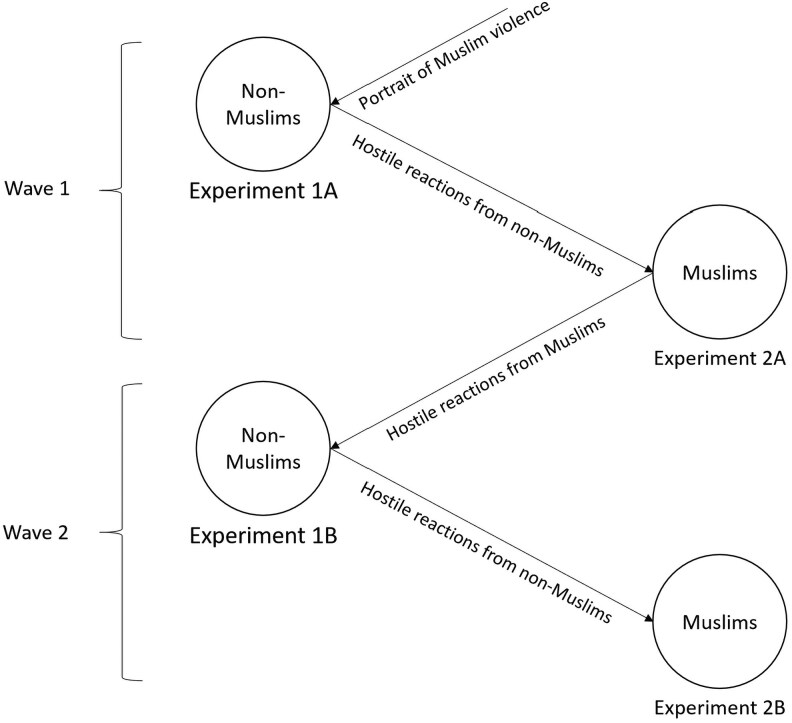
Illustration of the conceptual design and hypothesized reciprocal and escalating hostility between non-Muslim and Muslim participants in Denmark, integrating psychological and sociopolitical factors in intergroup conflict escalation.

In experiment 1B, we hypothesized that the non-Muslims from experiment 1A would exhibit increased violent behavioral intentions when presented with information indicating that Muslims advocated violence against them, especially after these Muslims were exposed to negative sentiments from the non-Muslims. Specifically, we anticipated a noticeable surge in violent intentions from time 1 to time 2, reflecting a cycle of intensified hostility. In experiment 2B, we postulate that the contentious strategies employed by non-Muslims, such as heightened violent behavioral intentions, will prompt a similar contentious response from Muslims, thereby perpetuating the vicious cycle. A conceptual overview of the design is provided in Fig. 1.

## Methods

### Overview of participants and procedure

Applying experimental and longitudinal approaches across two waves, we recruited 349 non-Muslims and 245 Muslim individuals in Denmark. To recruit our participants, we implemented multiple recruitment strategies. For experiments A1 and A2, non-Muslim participants were recruited through university Listservs, Facebook groups, and email networks. Given the challenges associated with recruiting participants from Muslim communities in Denmark, participants for experiments 1B and 2B were recruited through outreach to community leaders and snowball sampling. All participants were compensated. The study involved four survey experiments, in which participants were presented with information based on previous experimental results to simulate reciprocal intergroup escalation. Full details of the participants, materials, and procedures are provided in the Supplementary material.

### Experiment 1A

The experimental manipulation in experiment 1A that provided the entry point to our test of the hypothesized “vicious cycle” processes (Fig. 1) reflected data collected in prior research on violent extremism in Denmark and Sweden (25). Specifically, we manipulated intergroup threat to non-Muslim participants (members of the majority group) by presenting them with information either about the experiences and orientations of Muslims in Scandinavia (threat condition) or about a topic unrelated to intergroup relations (control condition). In the threat condition, participants read information that communicated non-technically the basic findings of Obaidi et al. (25). Muslim participants in Obaidi et al. (25) endorsed increased acts of violence against non-Muslim Danes and Swedes when they learned that Muslim traditions and norms were perceived as “backwards.” In other words, Muslim participants’ greater support for violence was cast as reactive, but it was a trigger of aggression, in line with certain political rhetoric in Denmark (44). We hypothesized that non-Muslims would react with violent intentions of their own, taking a critical step of escalation in the vicious cycle of intergroup hostility.

The ethical review board at the first author's institution approved the study at the time the research was conducted (protocol number: IRB15-0301) by the Committee on the Use of Human Subjects at Harvard University. All participants provided informed consent electronically before beginning the study; only those who consented were able to proceed to the survey.

In our study, the control condition was designed to provide a baseline comparison to the experimental manipulation while remaining unrelated to the specific intergroup context. Due to constraints on sample size and the challenges involved in recruiting participants from the minority population, we opted for a neutral text that matched the length of the experimental text but focused on an unrelated topic. While alternative control conditions could have been employed—such as presenting neutral or positive information about the other group—existing research suggests that even neutral information about a rival group may be perceived as threatening under intergroup threat conditions (27). Similarly, a positive control condition might confound the effects, making it challenging to discern whether observed differences stem from the experimental condition's negativity or the control condition's positivity. Due to these challenges, as well as statistical power considerations, we chose a neutral, nonintergroup-focused control condition.

#### Ethical considerations and debriefing procedures

The experimental manipulations in our research aimed to reflect existing rhetoric found in public discourse and political communication that participants are regularly exposed to in the Danish media, political speeches, and public debate. The information provided in the experimental conditions was based on prior empirical findings (25) but was adapted to include elements commonly found in public anti-Muslim rhetoric at the time, as documented in Danish political and media landscapes (44). This adaptation was intended to enhance the ecological validity of the study while investigating real-world escalation dynamics.

We recognize the ethical concerns of presenting participants with potentially exaggerated or distressing information. To address these concerns, participants were carefully debriefed after completing the study. The debriefing process included (i) clarifying the experimental manipulations and their purpose, (ii) correcting any misconceptions about the groups in question, and (iii) providing participants with resources for further support, if needed. Importantly, we received no complaints or expressions of distress from participants following the study. To mitigate the risk of leaving participants with elevated biases or hostility, the debriefing also emphasized intergroup relations’ complexity and multifaceted nature, aiming to foster a balanced understanding.

#### Participants

Participants in experiment 1A were 349 (133 = men, *M*_age_ = 31.57, SD = 11.42) non-Muslim Danes recruited for wave 1 of a two-wave sequence (the second being experiment 1B; see also Fig. 1). Data were collected through university Listservs, Facebook groups, and email networks (e.g. Copenhagen, Aarhus, and Aalborg) and from email Listservs at two-time points starting mid-July 2018. Participants received a gift card of 50 DKK (roughly equivalent to US$8) to an online fast-food chain (just-eat.dk). See Tables S1–S4 for information about the sample demographics.

We used power analysis to help determine our desired sample size. We were uncertain about the size of interaction between the manipulation and time, so instead we conducted a power analysis on the simple effect of the manipulation at one time point. As guidance, we used a meta-analysis on symbolic and realistic threats (27), suggesting that effects on intergroup outcomes are in the range of *r* = 0.40 to 0.45, or equivalent Cohen's *D*s exceeding 0.80. However, we considered the possibility that these estimates are upward biased (which Riek et al. also acknowledge as possible), and research, suggesting that replications often generate effect sizes about half the size of the original findings (45–47). As such, we considered a scenario where the experimental effect would be about half of the meta-analytic estimate (i.e. *D* = 0.40). Thus, our target *N* of 200 follows from a calculation in Gpower (48) for an independent, two-tailed t test with *α* = 0.05, a power of 0.80, and Cohen's *D* = 0.40.

As specified in the preregistration, we aimed to target a sample size of ∼200 participants. However, to account for attrition between waves 1 and 2, the power analysis focused on the experimental contrast in the second wave, with the goal of collecting a larger sample if possible. We recruited a considerably larger sample based on the potential for at least 30% of participants to drop out from wave 1 to wave 2 (49) and also because our recruitment method (using snowballing) did not allow us to prespecify an exact number of participants, as typically done on platforms like Prolific and Amazon Mechanical Turk. As such, we ended up with a sample of 349.

#### Procedure

Principal procedures, hypotheses, measures, analyses, and sample size estimations were preregistered at https://aspredicted.org/eh8dd.pdf. Deviations and ambiguities in the preregistrations are highlighted throughout the Methods and Results sections. All materials, code, and data for this and the remaining experiments are available at https://osf.io/6594b/?view_only=9627a2251d254f8382a9ab3874991c26. Additional data can be provided upon request. The participants in experiment 1A were randomly assigned to a threat condition or a control condition. In the threat condition, participants were asked to read a brief text summarizing findings from two previous studies conducted among Muslims in Denmark and Sweden (based on (25)). Specifically, participants read that Muslims in Scandinavia expressed increased violent intentions and endorsed the use of terroristic violence against the West after reading about a scenario in which non-Muslim Danes and Swedes expressed anti-Muslim views, such as that the Islamic religion, traditions, and norms were backward and incompatible with the Danish and Swedish way of life. The manipulation text includes language such as, “After reading this summary, we asked native Danes about their attitudes toward Muslims. Those who read the summary expressed hostile attitudes, including supporting and endorsing violence and expressing Islamophobic views toward Muslims.” Notably, the information provided was true at its core, but it was intentionally exaggerated to mirror the rhetoric commonly found in right-wing media, aiming to reflect the real-world amplification and evoke the perceived threat such rhetoric can elicit. The text representing the manipulation aimed to tap into safety threats (e.g. greater reactive support for terroristic violence) posed by Muslims against non-Muslims (see Supplementary material for complete materials in the manipulation). Participants in the control condition were asked to read a brief summary of a (fictional) study about how important it was to wear eyeglasses while driving (i.e. a neutral text matched in length to the experimental text).

After the manipulation, all participants completed a manipulation check (see Measures; see also (25)). In our analyses, we mainly focused on the dependent variable that was validated among violent extremists (42) and measured in the same way among non-Muslims and Muslims: violent behavioral intentions.

In the non-Muslim sample, we included additional measures assessing hostile attitudes commonly observed in majority groups. Specifically, in the Supplementary material, we report results for Islamophobia (50) and the Posse scale for ethnic persecution (29).

As indicated in our preregistered materials, we also included items on differential immigration policies, intergroup contact, and preferences for ingroup and outgroup members. However, these measures were based on categorical response options and did not capture the continuous dynamics central to hostility escalation. While they reflect other types of negative intergroup orientations, they do not directly assess violent intentions and thus fall outside the scope of the current work. Furthermore, the categorical response format limits their suitability for detecting gradual or quantitative changes in levels of negative orientations. For these reasons, we did not include these measures in the analyses reported here. The data are publicly available on Open Science Framework (OSF).

#### Measures

##### Manipulation check

To reduce the likelihood that participants would discern the main focus of the research on violent intentions, we assessed threat perceptions using items that did not mention physical threats. The threat perception measure consisted of five items (e.g. “Muslims hold values that conflict with the values of people like me,” see (29), *^α^*_T1_ = 0.90, *^α^*_T2_ = 0.91). Items were anchored at (1 = strongly disagree, 7 = strongly agree). For this and the subsequent experiments, all items were translated into Danish and subsequently back translated into English by two native Danish research assistants. For all measures included in this and subsequent studies, see Table S5.

### Violent behavioral intentions

A seven-item scale measured participants’ behavioral intentions to commit violence in defense of non-Muslims (e.g. “If nothing else helps, I’m prepared to use violence to defend non-Muslim Danes,” see also (29, 42), *^α^*_T1_ = 0.81, *^α^*_T2_ = 0.88). Items were anchored with 1 = strongly disagree and 7 = strongly agree.

### Experiment 2A

#### Participants

After conducting experiment 1A among non-Muslims, experiment 2A tested the response among a sample of Muslims. Here, too, participants were recruited as part of a two-wave sequence (experiment 2B represents wave 2; see also Fig. 1). Based on previous studies and our own experience, standard sampling methods produce extremely low response rates within Muslim communities due to distrust in research studies (51). Therefore, we employed two alternative recruitment strategies to facilitate participation: (i) contact with key community leaders to facilitate access to the target population (52) and (ii) a snowball sampling method. First, we reached out to well-respected religious and nonreligious members of Muslim networks in Denmark, including spokespersons for various Muslim communities, the head imams for mosques in three major cities (Copenhagen, Aarhus, and Aalborg), and the presidents of the largest Muslim associations. Relying on our network of trusted leaders, we established a culture of trust to distribute the online survey via email Listservs to Muslim minorities in Denmark. The participants were also encouraged to share the survey with their communities (snowball sampling). Second, we posted the link to our online survey in Facebook groups for Muslims living in Denmark.

Data were collected starting mid-October 2018. A convenience sample of 245 Muslim participants (sample 2) was recruited (115 = men, *M*_age_ = 23.12, SD = 12.11). As in experiment 1A, participants received a gift card of 50 DKK (∼US$8) to *justeat* (online fast-food chain). For clarity, although we hoped for a sample size on par with the non-Muslim sample, this was not feasible in practice.

#### Procedure

To examine escalating hostile reactions and intentions among Muslims in response to what non-Muslims had done, we followed the same procedure as in experiment 1A, with a few modifications. We replaced the priming manipulation used in experiment 1A with a summary of the results from experiment 1A conducted among non-Muslims (sample 1) to prime Muslims with threats and subsequently measure violent intentions. Specifically, participants were provided with information about non-Muslims’ hostility when they were exposed to information about Muslims’ endorsement of violence. The manipulation text includes language such as: “After reading this summary, we asked native Danes about their attitudes toward Muslims. Those who read the summary expressed hostile attitudes, including supporting and endorsing violence and expressing Islamophobic views toward Muslims.” Thus, we examined whether a group of Muslim participants would react with an endorsement of violence in response to learning about how a group of non-Muslims had, in fact, reacted that way (i.e. the results from experiment 1A).

#### Measures

##### Manipulation check

Participants were asked to complete a measure of threat perceptions consisting of five items, similar to those in experiment 1A (e.g. “Non-Muslim Danes hold values that conflict with the values of people like me,” see (29), *^α^*_T1_ = 0.87, *^α^*_T2_ = 0.94). Items were anchored with 1 = strongly disagree and 7 = strongly agree.

##### Violent behavioral intentions

A seven-item scale measured participants’ behavioral intentions to commit violence in defense of Islam and/or Muslims (e.g. “If nothing else helps, I’m prepared to use violence to defend Muslims,” *^α^*_T1_ = 0.85, *^α^*_T2_ = 0.92 (29)). These items were also anchored with 1 = strongly disagree and 7 = strongly agree.

### Experiment 1B

#### Participants

To examine escalating hostility over time, experiment 1B constituted a follow-up experiment among the non-Muslim Danish participants who participated in experiment 1A.

Of the 349 original participants, 154 took part in this second wave (i.e. 56% attrition rate). Although this is substantial attrition, we used Full Information Maximum Likelihood to mitigate these issues, at least to some extent (53). Perhaps more importantly, although the results may deviate from how an average person in Danish society would respond to the manipulated information, they could still capture an effect in the most relevant subpopulations. More specifically, just as the wish to stay in this study may differ between those who like or dislike its contents, the same is true in real life: Those who do not like the political rhetoric we echoed here may similarly disengage from media espousing it. Put differently, the most relevant population for generalization might not be the average Dane, but those in real life who self-select to continued exposure to the type of information that we manipulate here. Ideally, predictors of disengagement in real-life situations and these experiments should be compared; however, this is challenging to do in practice and is beyond the scope of this study.

Approximately half of the participants were in the threat condition, and the other half were in the control condition. Participants were re-contacted via email.^a^ Data for this study were collected beginning in February 2019. Those who participated in experiment 1B received an additional gift card for a fast-food chain, but this time for 100 DKK (∼US$16). When analyzing the data, we used inconsistent responses to certain demographic questions to screen for inattentive responses. Specifically, we excluded 12 participants who reported a different gender and/or age^b^ in experiment 1B compared with experiment 1A.

#### Procedure

The procedure of experiment 1B was identical to that of experiment 1A with one critical difference: here, we used the results from experiment 2A for priming the experimental group of non-Muslims with new information about increases in Muslim violent intentions. That is, those who had originally been assigned to the experimental condition now learned about the Muslim sample (experiment 2A) and, in particular, how some had responded with increased violent intentions toward non-Muslims. The stimulus presented to participants contained statements such as, “When these Muslims read about the concerns that native Danes had, they generally did not condemn the support for Jihadism in their communities … many reacted with even more hostility and increased support for Jihadist violence in Denmark and elsewhere in Europe.” More broadly, experiment 1B marked the start of the second cycle of our hypothesized vicious cycle, fueled by the hostile reactions observed in experiment 2A, in turn building the reactions in experiment 1A. The manipulation text is described in its entirety in the Supplementary material.

### Experiment 2B

Experiment 2B marked the completion of a second cycle of our hypothesized vicious cycle, and it was the final data collection. It followed the same logic as experiments 1B and 2A: presenting participants with information about escalated hostility observed in the preceding experiment in the non-Muslim sample (Fig. 1). The manipulation included the following phrasing: “When these Danes read about the concerns that Muslims in Denmark had, they generally did not condemn the support for violence against Muslims … many reacted with even more hostility toward Muslims in Denmark and elsewhere in Europe.”

Experiment 2B assessed counter negativity, illustrating that non-Muslims’ contentious tactics (e.g. increased violent intentions) encouraged a contentious response from Muslims, completing the vicious cycle.

#### Participants

Here, we invited the Muslim participants in experiment 1A to a follow-up study through email. Of the 245 participants who completed the first experiment, 107 returned to this second wave (i.e. experiment 1B). As in the non-Muslim sample, the attrition rate was 56% (for more discussion about attrition, see the Methods section for the non-Muslim sample). Half of the participants were in the threat condition, while the other half were in the control condition. Data for this study were collected beginning in June 2019. As in experiment 2A, participants in experiment 2B received a gift card of 100DKK (∼US$16) for a fast-food chain for this part of the research. Finally, parallel to what we did in the majority sample, we excluded participants in this sample who reported a different gender and/or a different age^c^ in experiment 2B compared with experiment 2A.

### Statistical analyses

To test the main predictions about increased hostility over time in the threat conditions, we ran parallel multi-level regression analyses for both participant groups (the non-Muslim and the Muslim samples) to examine the effects of the wave at level 1 (wave 1 vs. wave 2; within-subject) and the manipulations at level 2 (experimental vs. control; between subjects), as well as their (cross-level) interaction. The independent variables were dummy coded (wave 1 and control condition = 0; wave 2 and experimental condition = 1). Specifically, the effect from waves 1 to 2 was modeled as a random slope, influenced by the manipulation at level 2 (i.e. trends over time moderated by the manipulation). We used Full Information Maximum Likelihood estimation to account for missing values (i.e. model-based solution instead of imputations, which provide “best guesses” of what the results would have been, or rather what they would most likely be in the broader population, if there were no attrition).

All analyses were conducted in Mplus (version 7.3 (54)), except for manipulation checks, descriptive statistics, and bootstraps of those results, which were produced in SPSS (version 29, IBM) and JASP (version 0.19.3).

The outcome variables (primarily violent intentions) were heavily skewed and non-normal with an excess of minimum scores (i.e. most people indicating minimal violent behavior intentions; see Supplementary material for observed distributions). Thus, we sought to estimate a model that could account for such a distribution. For the main analyses, we used a zero-inflated Poisson distribution after a transformation to get integer data (based on the mean score of violent intentions × 7 [number of items],—7, to get integer data and to bring the minimum score to zero). Poisson models, developed for count data, might seem an unnatural choice with our outcome measure. Still, it could fulfill the goal of appropriately handling the spike of responses at the low end, and it has some advantages over other alternatives. For instance, log transformations would not solve the distributional problem (all minimum scores would just be recoded to the same, new score). Tobit regression is another option, but it comes with assumptions that the minimum scores are merely truncated, not real, and that the underlying variable (violent intentions here) is normally distributed. While that is possible, it is also quite possible that minimum scores are real, such that some people would not use violence under any circumstances, consistent with the theoretical meaning of pacificism. Had we observed behavioral acts of violence, it would be reasonable to expect a lot of genuine zeros in this (count) distribution (55, 56). Moreover, just as personality ratings may correspond to average behavioral trends (57), it is not unthinkable that people report violent intentions based on frequencies (i.e. counts) of past behavior. A reader would not have to go along with that speculation; the broader point is that any assumption about the true nature and generating process of the data (about truncation vs. genuine real minimum scores, and the normality of the latent distribution) is equally speculative. More importantly, from a pragmatic viewpoint, the real question is what kind of model fits the data best while imposing the least amount of analytic complexity and nonessential assumptions. For instance, hurdle models and sample selection models impose additional assumptions and complexity, without necessarily improving the model fit or easing interpretations of our results. From a pragmatic (Occam's razor) perspective, then, a Poisson model has considerable appeal (58, 59). Additional zero-inflation improved the model fit to our data while producing converging and replicable results across our datasets. The same was not true for other, more complex models.

For instance, we also tested a zero-inflated negative binomial model, which fits the data even better in the non-Muslim sample; however, this produced unreliable estimates when we applied the same model to the Muslim sample. As we sought to have parallel models in the two samples to optimize comparability, we proceeded with zero-inflated Poisson models in both cases. We also present results from simpler, regular Poisson models when estimating simple slopes (effects over time in each condition and sample), as these might be better suited for the observed distributions (Figs. S1–S10). Still, we believe the zero-inflated Poisson models are most appropriate, overall, even though the simpler models provide better support for our hypotheses (see Results).

Finally, although linear regression would be inadvisable given the distribution of data, we include results from such a model in the Supplementary material. These additional linear regression analyses are reported because we did not preregister the use of a more unconventional Poisson model for this type of data. Instead, we decided to use the Poisson model after seeing the actual distribution of responses, which indicated that the Poisson model was more statistically appropriate. Therefore, the results of the linear regression are provided in the Supplementary material to show that our findings do not hinge on our choice of the zero-inflated Poisson model as the more appropriate one for our main analyses. The results of the linear regression model, in fact, also offer clear support for our hypothesis. We reiterate that because of the highly skewed, non-normal distributions of the dependent measures, we do not believe linear regression is the correct way to perform inferential statistics on these data. We report the results of the linear regression analyses solely to be transparent and to show that our main analyses are not chosen on the basis of offering the best results.

## Results

Conceptually and methodologically, all experiments were linked such that each manipulation (except in experiment 1A) was based on the results from the preceding experiment in the other group (i.e. the non-Muslim vs. Muslim sample). Analytically, the proof of a vicious cycle of escalating intergroup hostility stems from showing that the effect of the manipulation (highlighting increased violent intentions in the other group) grows stronger from wave 1 to wave 2 in each group, respectively. We did not conduct an omnibus analysis for both samples, as the manipulations and measures were not perfectly parallel in the non-Muslim and Muslim samples (see Methods for details).

### Main analyses for the non-Muslim sample (experiments 1A and 1B)

As described under Statistical analyses, we used a multi-level regression model to test our predictions. The manipulation × wave interaction effect would capture our hypothesized vicious cycle that endorsement of violence gets worse over time when people learn that the other group endorses more violence (experimental condition) but not otherwise (control condition). We expected both groups to show this trend.

**Fig. 2.**
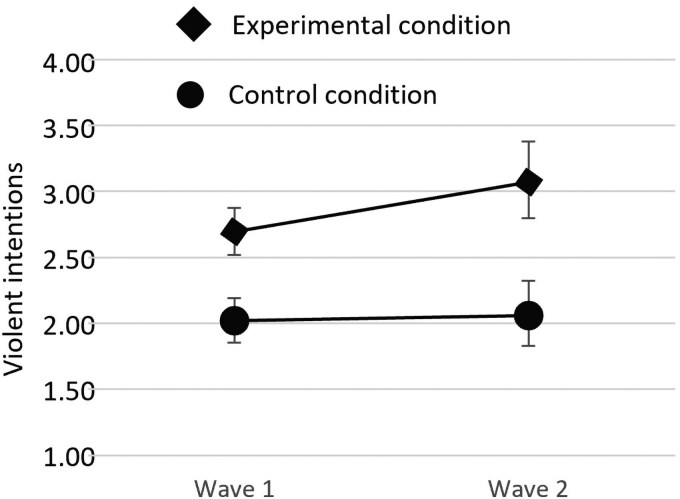
Mean levels (95% CI) of violent intentions in each condition and wave among non-Muslims. The wave 1 data are not from a premanipulation assessment, but rather follow the initial manipulation (i.e. a means difference is expected already at wave 1, which should grow in magnitude to wave 2; see the main text for details). CIs are based on 5,000 bootstrap samples.

Among non-Muslims, the effect of the manipulation alone was significant, *B* = 0.26, *P* = 0.005, and so was the predicted manipulation × wave (escalated hostility) interaction, *B* = 0.31, *P* = 0.019. Follow-up analyses for this interaction indicated that the simple effects of wave (i.e. increase in endorsement of violence over time) were significant in the experimental condition, *B* = 0.18, *P* = 0.014, but not in the control condition, *B* = −0.05, *P* = 0.665. Using a regular (not zero-inflated) Poisson model, the interaction was similar (*B* = 0.34, *P* = 0.032), and so were the simple slopes (experimental condition *B* = 0.20, *P* = 0.009, control condition *B* = 0.02, *P* = 0.872). Figure 2 presents a visualization of the interaction, displaying the mean levels of violent intentions across conditions and waves (note that this is for visualization only; the inferential test above is based on the multi-level zero-inflated Poisson regression described earlier).

### Main analyses for Muslims (experiments 2A and 2B)

Here, we ran another multi-level regression model with the exact same setup as the non-Muslim sample. Among the Muslim participants, there was no main effect of the manipulation on violent intentions, *B* = 0.10, *P* = 0.420. More importantly, however, we observed significant manipulation × wave interaction, *B* = 0.48, *P* = 0.013, replicating the effect from the non-Muslim sample. However, the breakdown of the interaction was a bit different.

**Fig. 3.**
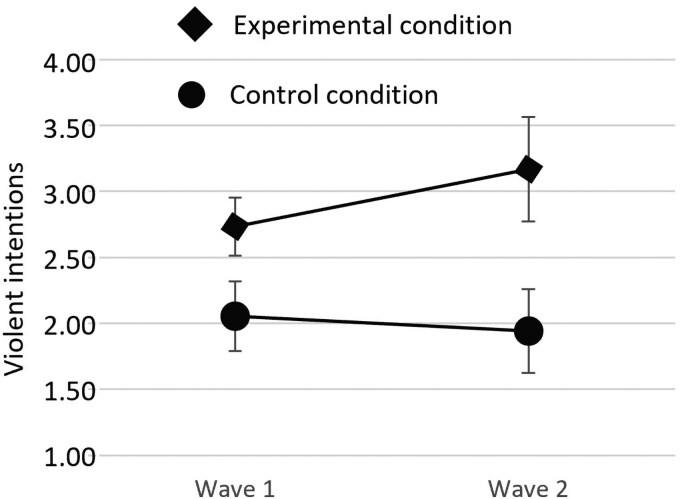
Mean levels (95% CI) of violent intentions in each condition and wave among Muslims. The wave 1 data are not from a premanipulation assessment, but rather follow the initial manipulation (i.e. a means difference is expected already at wave 1, which should grow in magnitude to wave 2; see the main text for details). CIs are based on 5,000 bootstrap samples.

While violent intentions increased somewhat over time in the experimental condition, the slope was nonsignificant, *B* = 0.11, *P* = 0.188. In the control condition, we instead observed a nonsignificant decrease, *B* = −0.23, *P* = 0.086. In other words, this interaction captures two trends in opposite directions for the experimental and control conditions. Yet, the interpretation of the simple slopes was sensitive to distributional assumptions. While zero inflation was reasonable to consider in the control condition, that was less clear in the experimental condition (Figs. S1–S10). Without zero inflation, the slope in the experimental condition was significant and positive, *B* = 0.21, *P* = 0.008. The slope in the control condition was again nonsignificant and close to zero, *B* = −0.06, *P* = 0.711. Overall, the interaction was robust across different distributional assumptions (*B* = 0.69, *P* = 0.003 for regular Poisson), but the simple slopes were less so. In Fig. 3, we provide the mean levels of violent intentions across conditions and waves (as for non-Muslims, this is strictly illustrative, and the inferential test above is based on the multi-level Poisson regression described in the previous paragraph).

## Discussion

The primary objective of the current study was to systematically examine hypotheses concerning the dynamics of escalating intergroup conflict. We investigated these processes, using an innovative combination of experimental and longitudinal methods, focusing on the specific context of relations between non-Muslim people in Denmark (the majority group) and Muslim people in Denmark (a minority group). In both groups, we found significant interactions between the experimental condition and time, indicating that the effect of perceived threats and hostility from the other group grew stronger over time. The breakdown of simple trends over time in each condition was not as clear in the Muslim sample and warrants interpretive caution, but the broader pattern was rather consistent in both samples. Put differently, it is less clear that Muslims who learned about violent intentions from the majority increase their hostility in return, but it appears in both samples that compared with the control conditions, the effect of perceived hostility from the other group became stronger.

While social and political tensions exist to some degree between non-Muslim and Muslim people in Denmark (33, 48), neither non-Muslim nor Muslim participants indicated high levels of violent intentions toward the other group in the initial wave of the research (Muslims: *M* = 2.44, Mdn = 2.14; non-Muslims: *M* = 2.27, Mdn = 2.14, on a scale from 1 = strongly disagree to 7 = strongly agree with a neutral midpoint of 4). It is worth noting that the modal response overall was at the absolute minimum on our 7-point scale, indicating that most participants were strongly opposed to violence. The only exception, where the modal response deviated from the minimum, was among non-Muslims in the experimental condition in wave 2 (see Supplementary Materials for details). As such, these findings should not be taken to mean that either group was particularly prone to violence in an absolute sense. In a relative sense, however, there were non-trivial shifts in both groups, and especially in the majority group, violent intentions increased in the experimental condition. The effect of increased openness to violence indicates a momentum that previous research and theoretical work have indicated could contribute to violent conflict or reciprocal violent extremism (1, 2) among non-Muslim people.

Further, while mid-range answers can be ambiguous and open to interpretation (e.g. reflecting ambivalence, openness to persuasion, or disengagement), our prior research suggests that they still carry psychological meaning—they are not merely random or neutral. For instance, even among former Afghan individuals involved in Islamist-inspired violent extremism, average scores on the same violent intentions scale remain below the midpoint (42), mean ≈ 3.9 on a 7-point scale). Thus, in the context of violent escalation, an upward shift from baseline hostility toward the midpoint of the scale may reflect a meaningful psychological tit-for-tat escalation of violent intentions.

The present research significantly advances an understanding of intergroup conflict escalation by examining the dynamic between Muslim and non-Muslim populations beyond the initial triggering event. It provides evidence of a mutually reinforcing interplay—a full sequence in which non-Muslims reacted with hostility to Muslims’ hostility, and Muslims subsequently reacted with hostility toward non-Muslims. More generally, the current paper addresses some of the key methodological blind spots identified in research on violent extremism—namely, a lack of longitudinal and experimental studies to unpack the causal dynamics of reciprocal violent extremism (60, 61). Although experiments are widely recognized as important in research on violent extremism, they are, in fact, rarely implemented (60). Previous experimental studies have primarily relied on vignettes to manipulate threat perception (25, 43). In the current study, in contrast, we conveyed information from previously published studies (e.g. a summary of the results), and the description thereof closely mimicked real-life rhetoric in right-wing media and some politicians (62). For instance, Breitbart News (63) reported findings from a study on Islamist-inspired extremism (11) in a manner similar to what we presented to participants in the experimental conditions. In this light, ecological validity should be high in the current studies.

Apart from potentially greater ecological validity, the current studies also stand out from previous research in terms of methodology. Psychological research has traditionally relied on student samples from relatively accessible populations (64). In contrast, we focused on a group often positioned at the center of public debates about extremism, namely Muslims, who also represent a minority group in Denmark. Prior research suggests that members of Muslim communities are often skeptical of participating in surveys (51, 65), and this typically requires significantly more effort to build trust and recruit participants compared with more accessible samples, such as students. We believe these understudied samples are important for a more comprehensive understanding of violent extremism in Western societies, alongside the majority-population extremism that we also studied here.

In addition, we conducted multiple experiments, each directly dependent on the previous experiments that produced the expected effects (see wave 2 materials and procedures). In other words, this data collection was both pain staking and delicate, with many features that could jeopardize the whole inquiry (e.g. not reaching enough Muslims or one manipulation failing and then spoiling all subsequent ones). Ultimately, this methodology enabled us to document a vicious cycle of intergroup hostility in a distinctively effective way.

### Limitations and future directions

An important caveat about this research is that a vicious cycle of hostility may not apply equally well to all groups or all members of the same group. For instance, individuals with a tendency toward intergroup violence because of either personality (42) or social context (66) might be more prone to (re-)enter a vicious cycle and accelerate their violent intentions more rapidly within these cycles compared with people without these characteristics or with different social influences. In addition, the relationship between Muslims and non-Muslim Europeans was chosen for this study as a plausible candidate for seeing a vicious cycle effect (2, 67) but, obviously, it cannot be taken as representative of all intergroup relations. Future studies could address these gaps by considering how threat perceptions interact with individual-level variables that predict violent extremism.

Further, with respect to the intergroup context in the current research, it is important not to fall prey to stereotypes portraying Muslims, in general, as violent extremists. We note, for example, that in the current research Muslim participants did not seem to differ from non-Muslim participants in violent intentions at wave 1 or wave 2 in the control or experimental conditions. Future research might consider more fully how individuals with a tendency toward intergroup violence respond across different group contexts and whether such tendencies are shaped more by situational dynamics and personality traits than by group membership alone.

The nature of the information presented also moderates the degree to which a vicious cycle occurs. Our objective was to examine the dynamics of a hypothesized vicious cycle within a specific naturalistic intergroup context involving non-Muslim and Muslim participants in Denmark. To this end, we employed stimuli derived from actual research findings. We intentionally exaggerated to mirror rhetoric designed to trigger real-world amplification processes and contribute to escalating intergroup tensions within the proposed cycle. For example, in experiment 1A, non-Muslim participants in the threat condition read about research indicating that Muslims were more likely to endorse violence when their traditions and norms were perceived as backwards by non-Muslims (25). Describing seemingly unprovoked acts of violence by members of the other group—perceived as less justified or rational—could potentially accelerate the escalation of violent intentions even faster. Future research might systematically vary specific elements of the perceived threat in the response of the other group (e.g. magnitude, controllability, and justification) to further explore the dynamics of the vicious cycle and identify strategies to de-escalate the conflict.

Another question, both theoretically and practically significant, is how the citizenship status of Muslim participants might moderate violent intentions. While shared Danish identity could potentially mitigate the likelihood of conflict escalation among Muslim participants, previous research on violent extremism suggests that this effect may depend heavily on whether these individuals perceive themselves as equal and respected citizens within Danish society. Earlier studies have demonstrated that feelings of marginalization and subordination in Denmark and other Western nations can be linked to increased violent extremism among native-born Muslims (68).

We acknowledge the potential value of exploring multiple control conditions in future research to further disentangle these effects. By varying the content of control conditions—neutral, positive, or negative—researchers can deepen their understanding of the mechanisms driving intergroup hostility and escalation. For example, presenting participants with information about the rival group that is neither overtly negative nor positive could help clarify whether the observed effects are driven by the violent content or simply the mention of the rival group.

### Societal implications

Empirical and anecdotal accounts indicate that in tensions between Muslims and non-Muslims in Europe, both groups often see themselves as merely responding to outgroup hostility, rather than acting as aggressors (2). Similarly, whereas adversary groups perceive their own group's hostility as morally justifiable and motivated by ingroup love, they attribute the outgroup's hostility to hatred and moral degradation (69, 70). Hence, making each party aware of their own bias and the extent to which each party contributes to conflict escalation may be the first step toward promoting conciliatory attitudes and behaviors and ultimately leading to conflict resolution.

In addition, despite some evidence here of a reciprocal effect of hostility between Muslims and non-Muslims overall, the modal response was a minimum endorsement of each item on the violent intentions measure. Future studies could leverage this insight to reverse the cycle of hostility. Recent research has shown that correcting meta-(mis)perceptions (i.e. the belief that the outgroup endorses violence against the ingroup) can significantly reduce support for intergroup violence and increase opposition to it (71). Interventions that provide accurate information correcting the ingroup's meta-(mis)perceptions about the outgroup's violent intentions may serve as the first positive step toward breaking the cycle of hostility and de-escalating intergroup conflict and violence.

## Supplementary Material

pgaf338_Supplementary_Data

## Data Availability

All materials, code, and data for this and the remaining experiments are available at https://osf.io/6594b/?view_only=9627a2251d254f8382a9ab3874991c26. Language correction for this manuscript was assisted by AI tools. The authors take full responsibility for the content and interpretations presented in this study.

## Notes

a We included a link in the initial survey directing participants to a separate, unlinked email collection form to ensure anonymity. This allowed us to compile contact lists for each condition without knowing which survey response belonged to which participant. The same list was used to invite participants to the second wave. Importantly, at no point we were able to link individual email addresses to their responses in either wave, maintaining full anonymity throughout.

b We did not exclude participants reporting +1 years in the second session, as they could have had a birthday since session 1. We also examined inconsistent answers to a question about what party they had voted for in the last election. However, there was a surprisingly high frequency of mismatches here (>10% of the sample), and we suspect that participants may have misinterpreted this question as a matter of current voting preference. Thus, we did not use this variable for further exclusions, beyond reporting a different gender and/or age across the two sessions.

c Due to a much slower data collection when sampling from a specific population (i.e. Muslims in Denmark), some participants would be 2 years older in experiment 2B. Again, we accounted for this time lag and only excluded impossible age reporting (e.g. 32 in session 1 and 25 in session 2). The age variables also contained some coding errors, which we set aside when we examined mismatches across the experimental sessions.
