# Supplementary material for: Reciprocal escalation of violent extremism: Experimental and longitudinal evidence from Denmark
Source: PNAS Nexus. 2025 Oct 22;4(12):pgaf338. doi: 10.1093/pnasnexus/pgaf338 (PMC12673851; doi:10.1093/pnasnexus/pgaf338)
Supplement: pgaf338_Supplementary_Data [file pgaf338_supplementary_data.pdf]

Supplementary material for:

Reciprocal Escalation of Violent Extremism: Experimental and Longitudinal Evidence from Denmark

Overview of Demographic Variables

Table S1

Overview of the total sample size, age and proportion of female participants

Experiments	Samples	Wave	N	Females	Mean Age
1A	Non-Muslim	1	349	58.4	31.85
2A	Muslim	1	245	45.5	23.12
1B	Non-Muslim	2	154	41.6	30.36
2B	Muslim	2	107	54.5	40.23

Table S2

Self-reported socioeconomic status

Experiments	% upper	% upper middle	% middle	% lower middle	% working class
1A	2.6	33.9	48.6	12.8	1.9
2A	2.9	12.2	47.3	13.5	1.6
1B	6.0	28.9	47.0	15.4	2.0
2B	1.9	20.6	42.1	15.9	.9

Socioeconomic status was measured by asking participants to indicate which socioeconomic class they belong to: Upper class, Upper middle class, middle class, lower middle class, or working class. In Study 3, socioeconomic status was not measured.

Table S3

Self-reported education

Education	Experiments			
	1A	2A	1B	2B
Less than 7	.3	.8	.7	.9
High school student	1.9	6.5	.7	7.5
Completed high school	4.1	6.1	4.0	6.5
Student at technical or vocational school	1.3	2.9	-	2.8
Completed technical or vocational school	4.5	6.1	4.0	8.4
University student	36	17.1	35.8	20.6
Completed University with degree	22.6	16.7	25.2	14
Some post-graduate education	6.7	4.5	6.6	7.5
Post-graduate degree (e.g., MA, MS, PhD)	22.6	11.4	23.2	11.2

Table S4

Sefl-reported employment

Experiments	% full time	% part time	% student	% unemployed
1A	30.4	8.3	57.2	4.2
2A	28.6	10.2	33.1	2.9
1B	30.7	4.7	58.7	6.0
2B	24.3	9.3	36.4	2.8

The Experimental Vignettes, Experiment 1A, non-Muslims - Wave 1

Threat condition

In the following, we would like you to read the results of a study we conducted among Muslims in Denmark and answer a few questions. In particular, we wanted to investigate the extent to which Muslims support terrorism against Europe after reading about a fictional scenario. We presented our Muslim participants with the following (partly fictional) text:

"The majority of native Danes see the presence of Muslim immigrants in Denmark as a problem, and they see Islamic culture and religion as backwards. They see Islamic values, norms and traditions as incompatible with Danish values, norms and traditions. Because of this, native Danes believe that Muslim immigrants pose a threat to Denmark. Furthermore, the study shows that native Danes do not think that Muslims belong to Danish society".

After Muslims read this text, we asked them about their attitudes toward terrorism against Europe and against those who harm Muslims. Those Muslims who read this text expressed positive attitudes toward groups who committed acts of violence against Europe and against those who harmed Muslims. They were also supportive of groups who wanted to commit acts of violence in Europe.

Control condition

In the following, we ask you to read the results of a study conducted among Danes in Denmark and answer a few questions. This study aimed to investigate the extent to which Danes became more positively inclined toward using driving glasses after reading a short informational text about them. The Danish participants were presented with the following informational text:

"Most studies show that driving glasses increase road safety. Driving glasses are designed to make driving safer and more comfortable for drivers and other road users. They can enhance contrasts around the driver, and their anti-reflective coating can reduce glare from oncoming cars and strong sunlight. Driving glasses are no longer just for professional drivers, as everyone now has the opportunity to own multiple pairs of glasses."

After reading the informational text, the participants were asked about their attitudes toward driving glasses. Those who read this text expressed positive attitudes toward using driving glasses and were also more inclined to recommend them to other drivers.

The Experimental Vignettes, Experiment 2A – Muslims

Threat condition

We would like you to read the summary of the results of a study we conducted among native Danes in Denmark and then answer a few questions. The study aimed to investigate the extent to which native Danes support Islamophobic attitudes and violence against Muslims after reading the results of another study we conducted, which showed how some Muslims in Denmark endorsed violence against Europe and those who harmed Muslims. We presented our native Danish participants with the following summary:

"Studies show that, under certain circumstances, some Muslims in Denmark are willing to support and use violence against Europeans and those who harm Muslims."

After reading this summary, we asked native Danes about their attitudes toward Muslims. Those who read the summary expressed hostile attitudes, including supporting and endorsing violence and expressing Islamophobic views toward Muslims.

Control condition

In the following, we ask you to read the results of a study conducted among Danes in Denmark and answer a few questions. This study aimed to investigate the extent to which Danes became more positively inclined toward using driving glasses after reading a short informational text about them. The Danish participants were presented with the following informational text:

"Most studies show that driving glasses increase road safety. Driving glasses are designed to make driving safer and more comfortable for drivers and other road users. They can enhance contrasts around the driver, and their anti-reflective coating can reduce glare from oncoming cars and strong sunlight. Driving glasses are no longer just for professional drivers, as everyone now has the opportunity to own multiple pairs of glasses."

After reading the informational text, the participants were asked about their attitudes toward driving glasses. Those who read this text expressed positive attitudes toward the use of driving glasses and were also more inclined to recommend them to other drivers.

The Experimental Vignettes, Experiment 1B – non-Muslims - Wave 2

Threat condition

In the initial data collection for this study, we asked you and other native Danes about your attitudes toward Muslims in Denmark. Many who participated expressed concern about Muslims who support Jihadist violence in Denmark and Europe. This made us curious about how Muslims in Denmark would react to the findings from our native Danish participants, so we conducted another study with Muslim participants. When these Muslims read about the concerns that native Danes had, they generally did not condemn the support for Jihadism in their communities. They did not seem to have much awareness of the problems highlighted by the native Danish participants. In fact, when we tried to educate Muslims about the viewpoints of native Danes, many reacted with even more hostility and increased support for Jihadist violence in Denmark and elsewhere in Europe.

In this follow-up study, we want to make another attempt to better understand how native Danes perceive Muslims and the basis for these beliefs and opinions. In other words, we are interested in how you view Muslims in general and why you hold these views. We are also interested in your reactions to what we found in our study among Muslim participants.

Control condition

In the following, we ask you to read the results of a study conducted among Danes in Denmark and answer a few questions. This study aimed to investigate the extent to which Danes became more positively inclined toward using driving glasses after reading a short informational text about them. The Danish participants were presented with the following informational text:

"Most studies show that driving glasses increase road safety. Driving glasses are designed to make driving safer and more comfortable for drivers and other road users. They can enhance contrasts around the driver, and their anti-reflective coating can reduce glare from oncoming cars and strong sunlight. Driving glasses are no longer just for professional drivers, as everyone now has the opportunity to own multiple pairs of glasses."

After reading the informational text, the participants were asked about their attitudes toward driving glasses. Those who read this text expressed positive attitudes toward using driving glasses and were also more inclined to recommend them to other drivers.

The Experimental Vignettes, Experiment 2B - Muslims

Threat condition

In the initial data collection for this study, we asked you and other Muslims about your attitudes toward native Danes. Many who participated expressed concern about native Danes who support the violence against Muslims. This made us curious about how native Danes would react to the findings from our Muslim participants, so we conducted another study with native Danish participants. When these Danes read about the concerns that Muslims in Denmark had, they generally did not condemn the support for violence against Muslims. They did not seem to have much awareness of the problems highlighted by the Muslim participants. In fact, when we tried to educate native Danes about the viewpoints of Muslims, many reacted with even more hostility toward Muslims in Denmark and elsewhere in Europe.

In this follow-up study, we want to make another attempt to understand better how Muslims perceive native Danes and the basis for these beliefs and opinions. In other words, we are interested in how you view native Danes in general and why you hold these views. We are also interested in your reactions to what we found in our study among native Danish participants.

Control condition

In the following, we ask you to read the results of a study conducted among Danes in Denmark and answer a few questions. This study aimed to investigate the extent to which Danes became more positively inclined toward using driving glasses after reading a short informational text about them. The Danish participants were presented with the following informational text:

"Most studies show that driving glasses increase road safety. Driving glasses are designed to make driving safer and more comfortable for drivers and other road users. They can enhance contrasts around the driver, and their anti-reflective coating can reduce glare from oncoming cars and strong sunlight. Driving glasses are no longer just for professional drivers, as everyone now has the opportunity to own multiple pairs of glasses."

After reading the informational text, the participants were asked about their attitudes toward driving glasses. Those who read this text expressed positive attitudes toward using driving glasses and were also more inclined to recommend them to other drivers.

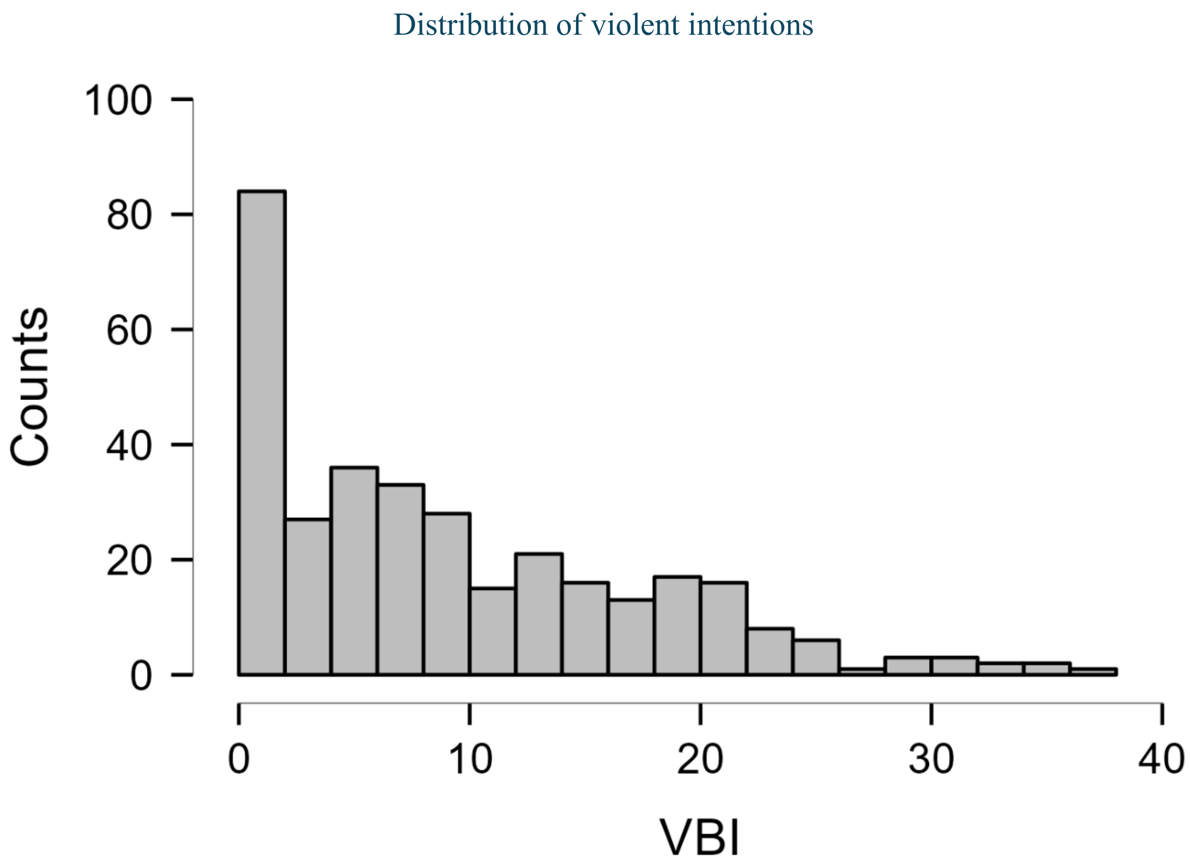

Figure S1. Distribution of violent intentions among non-Muslims, aggregated across waves and conditions (after scale transformation to integer data, see manuscript for details; subsequent figures per wave and condition are on the original scale).

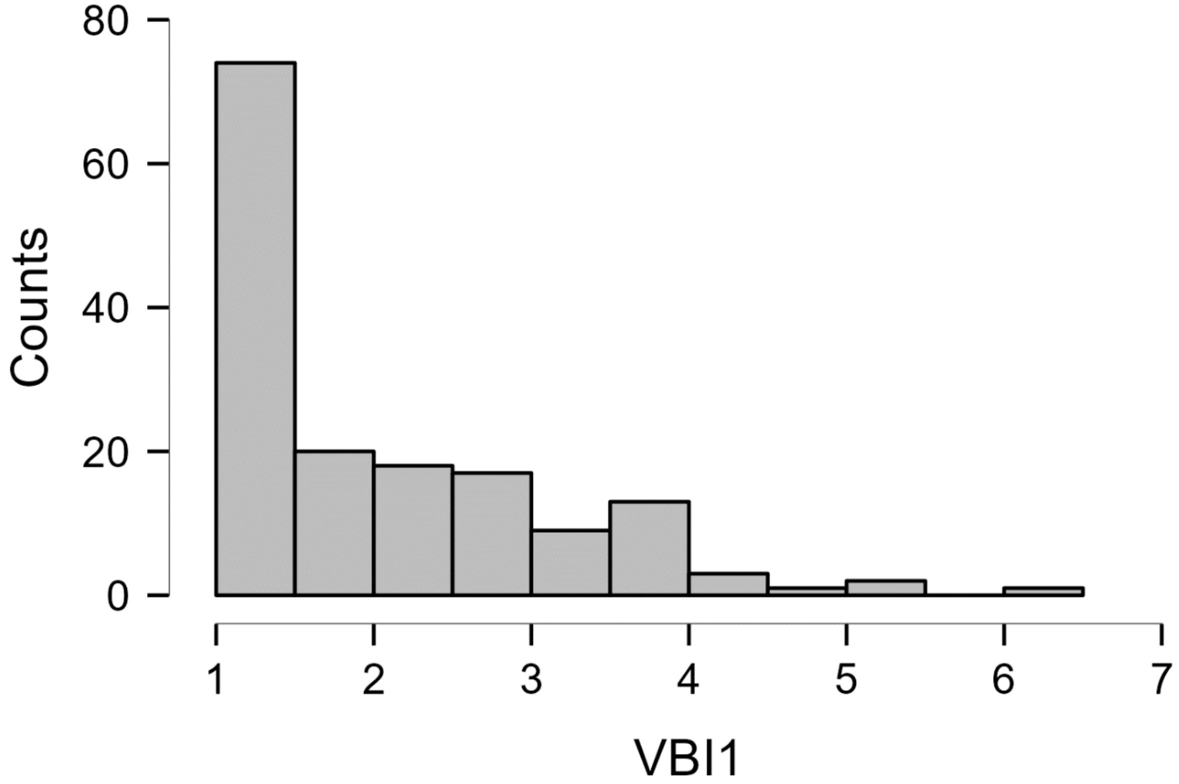

Figure S2. Distribution of violent intentions in Experiment 1A (non-Muslims), at wave 1 in the control condition.

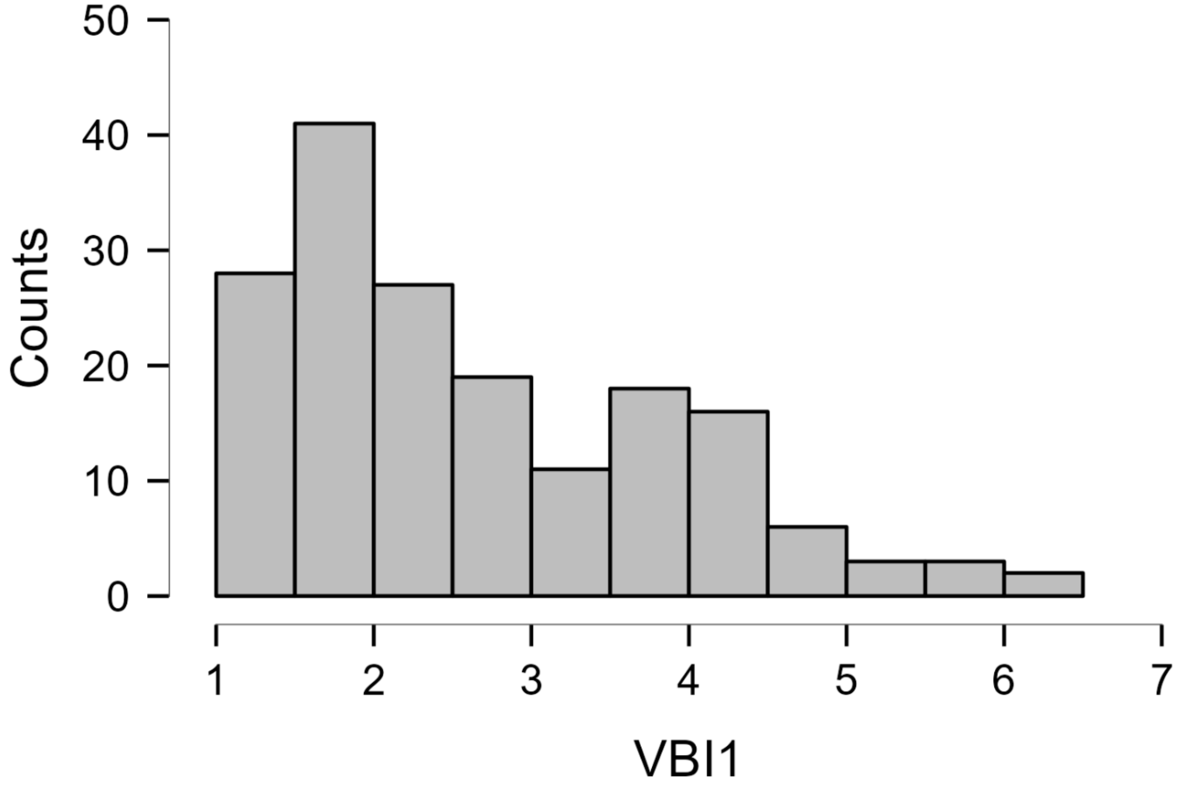

Figure S3. Distribution of violent intentions in Experiment 1A (non-Muslims), at wave 1 in the experimental condition.

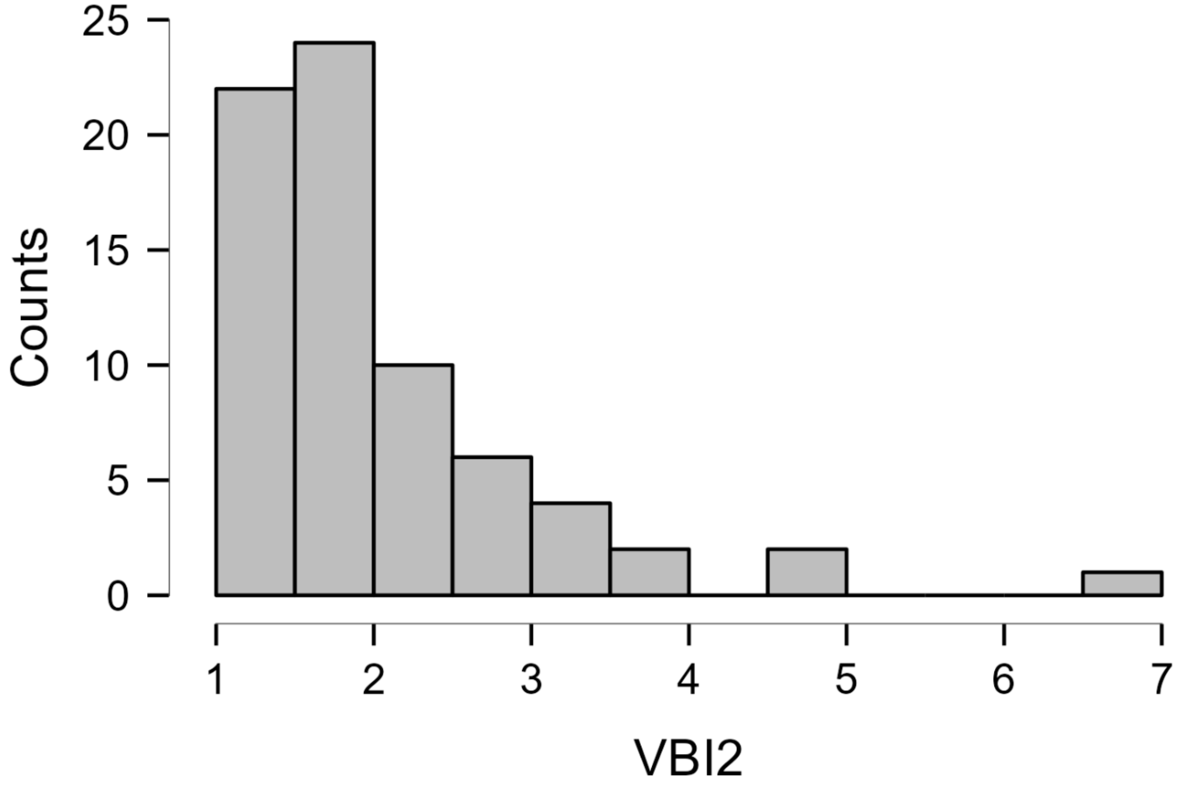

Figure S4. Distribution of violent intentions in Experiment 2A (Non-Muslims), at wave 2 in the control condition.

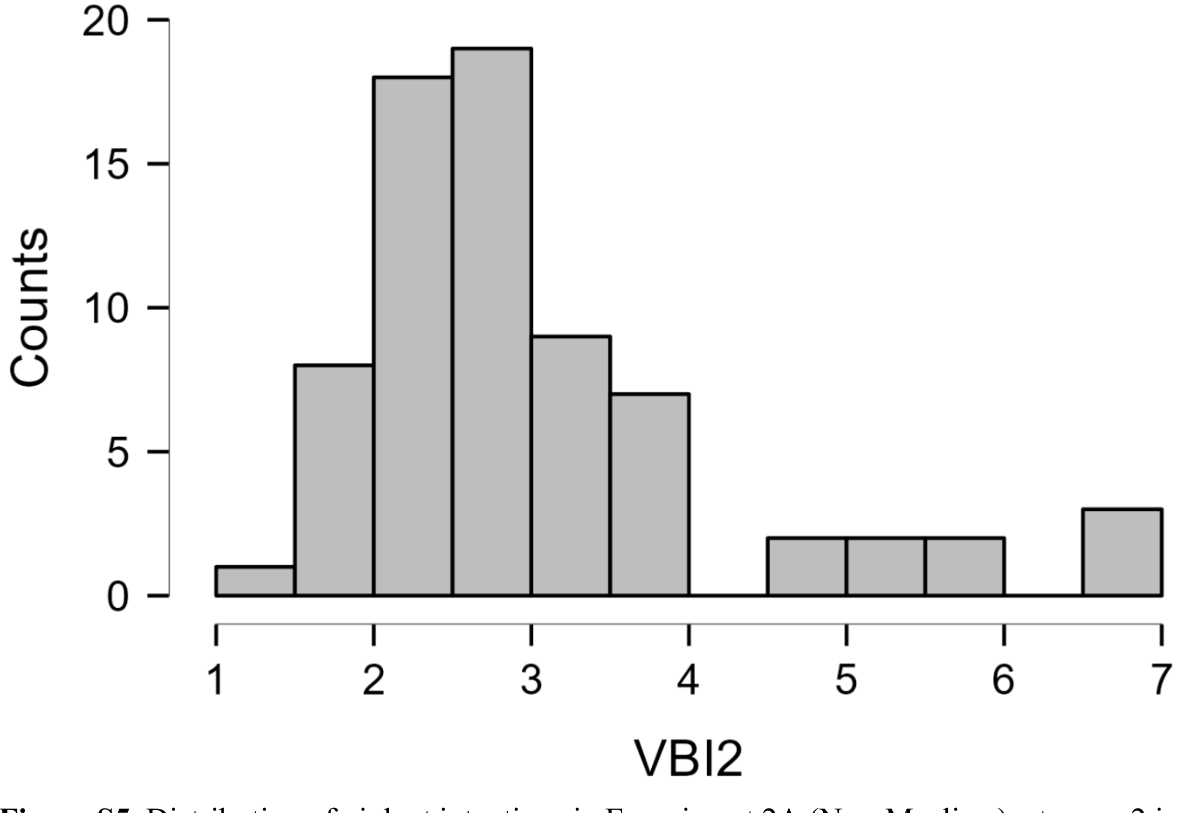

Figure S5. Distribution of violent intentions in Experiment 2A (Non-Muslims), at wave 2 in the experimental condition.

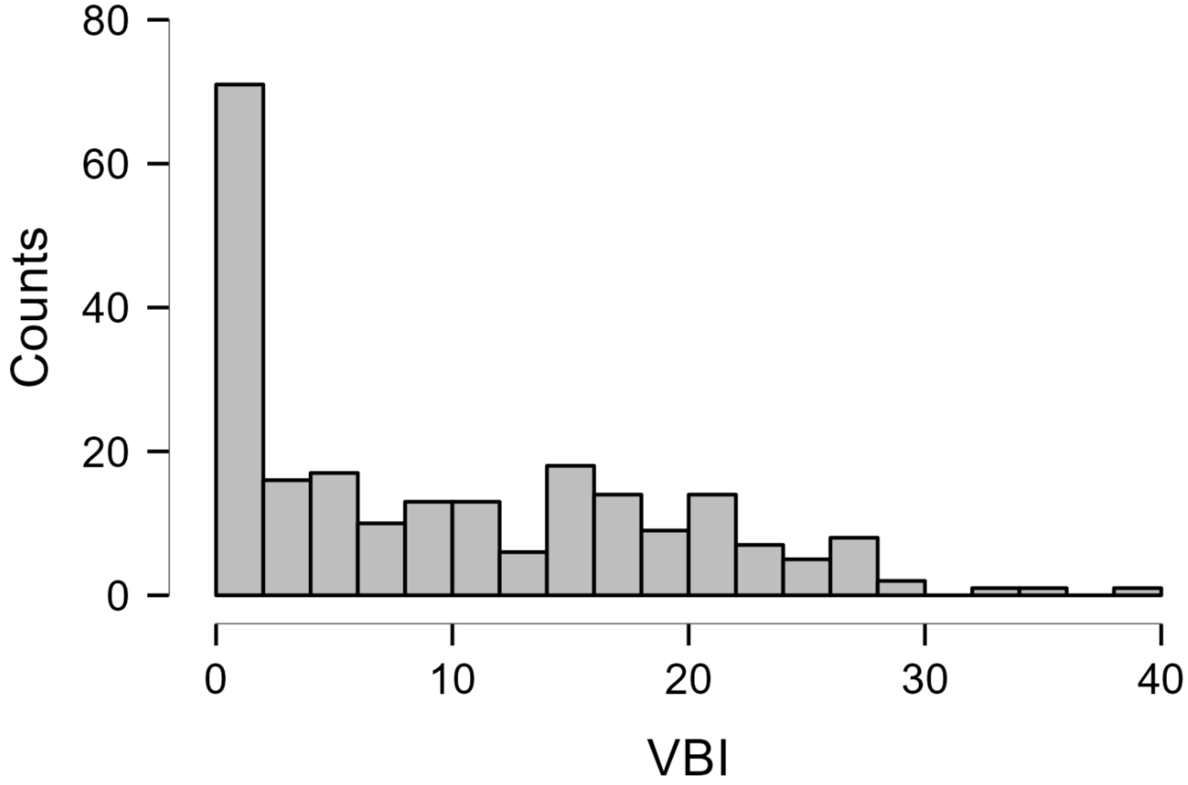

Figure S6. Distribution of violent intentions among Muslims, aggregated across waves and conditions (after scale transformation to integer data, see manuscript for details; subsequent figures per wave and condition are on the original scale).

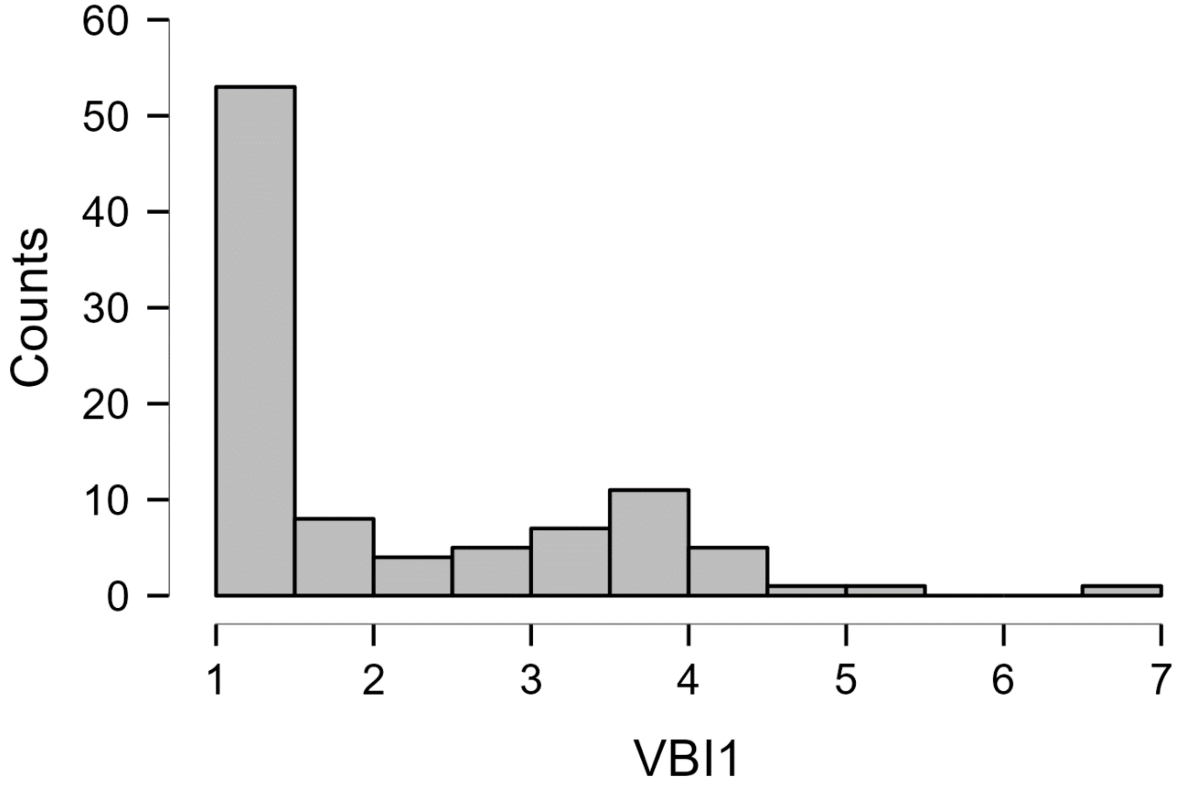

Figure S7. Distribution of violent intentions in Experiment 2A, at wave 1 in the control condition.

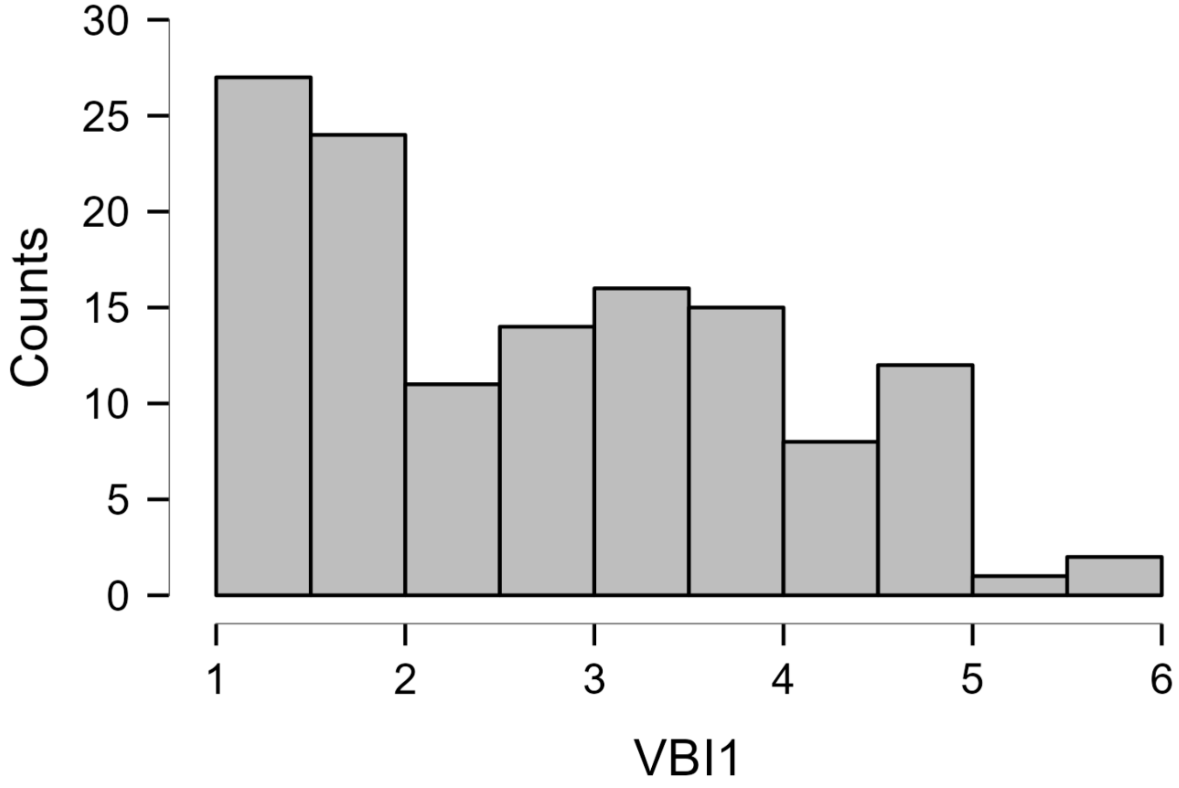

Figure S8. Distribution of violent intentions in Experiment 2A, at wave 1 in the experimental condition.

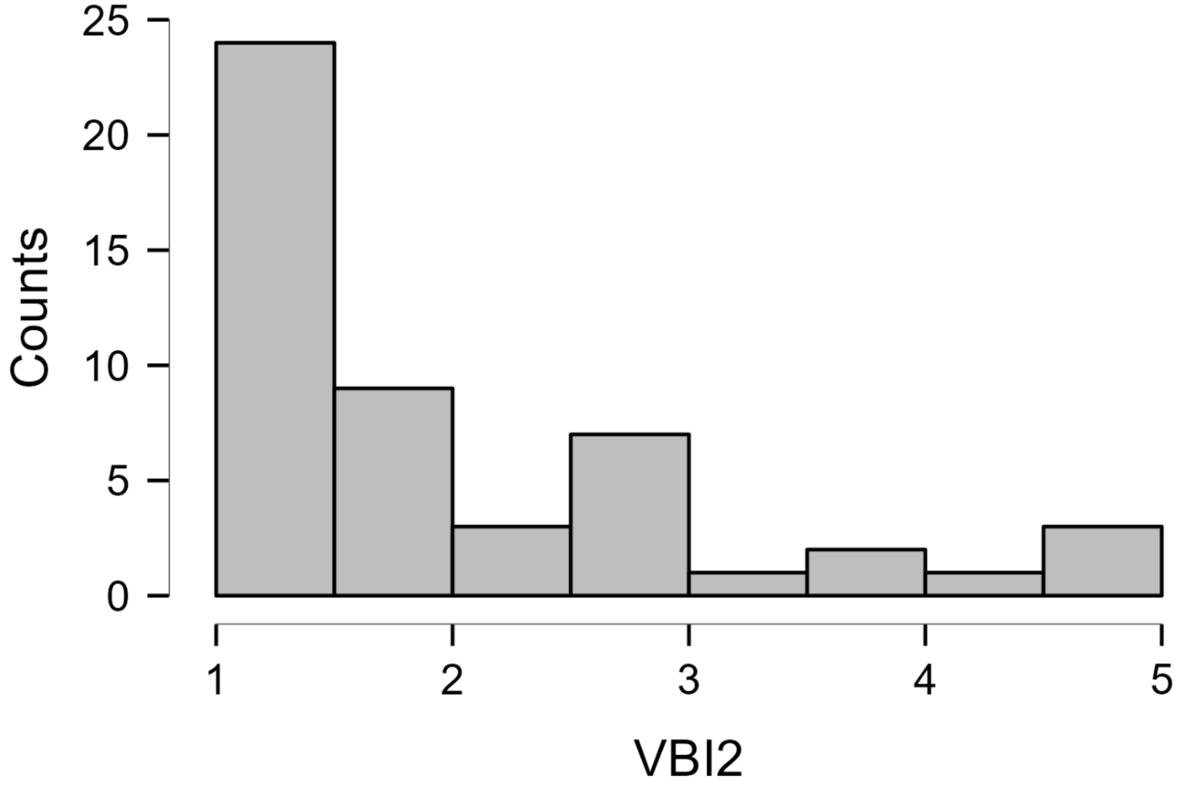

Figure S9. Distribution of violent intentions in Experiment 2A, at wave 2 in the control condition.

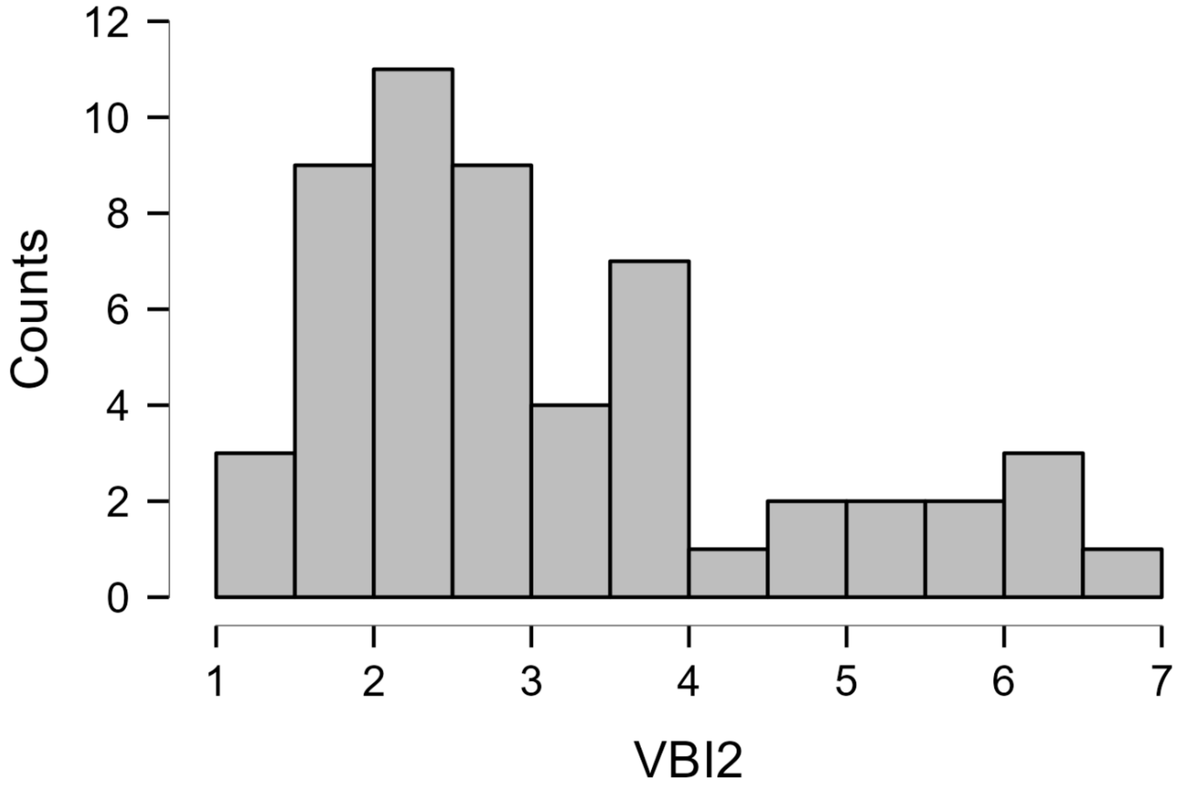

Figure S10. Distribution of violent intentions in Experiment 2A, at wave 2 in the experimental condition.

Measures and Items Included in Experiment 1A, 2A, 1B and 2B

Table S5

Measures and items

Immigration Policy Preferences by group characteristics
Do you think that legislations should be changed to make it easier or harder for the following groups to immigrate to Denmark?

1. White Muslims (e.g. from Bosnia).
2. Black Muslims (e.g. from Somalia).
3. Black Christians (e.g. from USA).
4. White Christians (e.g. from Germany).

<u>Cultural threat perception</u>

1. The lifestyle of Muslims/Danes poses a threat to the cultural and religious practices of Danes.
2. The family values of Muslims/Danes are not compatible with those of Muslims/Danes.
3. The identity of Danes/Muslims is threatened in Denmark.
4. Muslims/Danes have values that conflict with the values of people like me.
5. Muslims Danes threaten the way I live my life.

Islamophobia

1. I would support any policy that would stop the building of new mosques in the Denmark/Norway.
2. If possible, I would avoid going to places where Muslims would be.
3. I would become uncomfortable speaking with a Muslim.
4. Just to be safe, it is important to stay away from places where Muslims could be.
5. I dread the thought of having a boss that is Muslim.
6. If I could, I would avoid contact with Muslims.
7. If I could, I would live in a place where there were no Muslims.
8. Muslims should not be allowed to work in places where many Danes/Norwegians gather, such as airports.

Contact preference

If you could choose your neighbours, how strong is your preference for Muslims versus Danes?

1. If you could choose your neighbours, how strong is your preference for Muslims versus Danes?
2. If you had to go to a doctor, would you prefer the person to be Muslim or Dane?
3. Would you prefer your extended family member (e.g. your brother's wife or sister's husband) to be Danish or Muslim?

Behavioral Violent Intentions

1. If nothing else helps I'm prepared to use violence against Westerners to defend Muslims.
2. As a last resort I'm personally ready to use violence against Westerners for the sake of other Muslims.
3. I will personally use violence against Westerners harming other Muslims that I care about.
4. I'm ready to go and fight against Westerners for the sake of Muslims in another country.
5. I'm not prepared to use violence against Westerners in any situation. (R)
6. I will not personally use violence against Westerners to help Muslims. (R)
7. Even as a last resort, I will not use violence against Westerners for the sake of other Muslims. (R)

Willingness and Support of Muslim Persecution

Imagine that someday in the future the American government decides to outlaw Muslim organizations and requests all citizens to do their best to make sure that the law has a successful effect. Please indicate on a scale from 1 (strongly disagree) to 7 (strongly agree) how much you agree with the following statements.

1. I would tell my friends that it was a good law.
2. I would tell the police about any members of Muslim organizations that I knew.
3. I would help hunt down members of Muslim organizations and turn them over to the police.
4. I would participate in attacks on Muslim headquarters if supervised by the proper authorities.

5. I would support the use of physical violence to make members of Muslim organizations reveal the identity of other Muslims.
6. I would support the execution of Muslim leaders.

Note. (R) = Reverse scored.

Additional outcomes in non-Muslim sample

For the main analyses, we focused on the outcome that was the same for our Muslim and non-Muslim participants: violent intentions/willingness to defend one's group with violence (Anonymous). In the non-Muslim sample, however, we also had additional measures assessing hostile attitudes more commonly assessed in majority groups. Specifically, here we report results also for Islamophobia (Anonymous) and the Posse scale for ethnic persecution (Anonymous), considering some conceptual overlap with our main dependent variable. For clarity, we do not include results for threat perceptions, ingroup-outgroup preferences, differential immigration rules, and inter-group contact because (a) these measures are conceptually further from our focus here (violence and extremism) and will be reported elsewhere, and (b) because they have a different response format (i.e. could not be used to answer the principal question of this manuscript). However, data on the non-included variables are available on OSF and available to anyone interested in them. With that noted, we now turn to results for the Islamophobia and Posse instruments.

Like the measure of violent intentions, the Islampohopbia and Posse instruments had considerable skew, with most participants showing minimal agreement with the (rather extreme) statements. As such, we tested a zero-inflated Poisson and a zero-inflated negative-binomial model for each outcome (after transforming the responses to integers with a minimum of zero, as done with the violent intentions measure; see main text for details). The dispersion parameter was significant for Islamophobia (p = .019), suggesting that a negative binomial model would be preferable in this case. For the Posse scale, the dispersion parameter was non-significant (p = .09), suggesting that a Poisson model is more parsimonious. Still, we present both types of models for each outcome for transparency. Below are the Mplus output files, showing both the model specifications (identical to main analyses, except alternating between Poisson and negative binomial) and the results.

The key result – the cross-level wave × condition interaction – is highlighted in boldface.

Islamophobia, zero-inflated Poisson model

INPUT INSTRUCTIONS

```
  DATA:        FILE = Danish_majority.txt;

  [Note: variable list is the same as in the files on OSF and left out here for abbreviation.]

  USEVARIABLES =
  Subject
  ISLAMOPH
  WaveD
  CondD;

  WITHIN = WaveD;
  BETWEEN = CondD;
  CLUSTER = Subject;

  COUNT ARE ISLAMOPH (pi);
```

```
 MISSING ARE ALL (-9);
 USEOBSERVATIONS ARE (DoubleChosen EQ 0) AND (GenAgeMat EQ 0);

 ANALYSIS: TYPE = TWOLEVEL RANDOM;

 MODEL:

 %WITHIN%
 sISLAMOPH | ISLAMOPH ON WaveD;

 %BETWEEN%
 ISLAMOPH ON CondD;
 sISLAMOPH ON CondD;

 OUTPUT: SAMPSTAT TECH1;

 PLOT: TYPE = Plot3;

SUMMARY OF ANALYSIS

Number of groups                                                 1
Number of observations                                           440

COUNT PROPORTION OF ZERO, MINIMUM AND MAXIMUM VALUES

      ISLAMOPH    0.505        0        48


SAMPLE STATISTICS


     SAMPLE STATISTICS


           Means
              WAVED         CONDD
              ________      ________
 1              0.318         0.514


           Covariances
              WAVED         CONDD
              ________      ________
 WAVED          0.217
 CONDD         -0.004         0.250


           Correlations
              WAVED         CONDD
              ________      ________
```

```
 WAVED         1.000
 CONDD        -0.019        1.000


THE MODEL ESTIMATION TERMINATED NORMALLY

MODEL FIT INFORMATION

Number of Free Parameters                        6

Loglikelihood

          H0 Value                      -1304.145
          H0 Scaling Correction Factor     3.3871
            for MLR

Information Criteria

          Akaike (AIC)                   2620.289
          Bayesian (BIC)                 2644.810
          Sample-Size Adjusted BIC       2625.769
            (n* = (n + 2) / 24)

MODEL RESULTS

                                                    Two-Tailed
                    Estimate       S.E.  Est./S.E.    P-Value

Within Level

Between Level

 SISLAMOPH  ON
    CONDD             -0.692      0.247     -2.799      0.005

 ISLAMOPH   ON
    CONDD              1.082      0.206      5.241      0.000

 Means
    ISLAMOPH#1        -0.018      0.103     -0.180      0.857

 Intercepts
    SISLAMOPH          0.340      0.214      1.589      0.112
    ISLAMOPH           1.293      0.167      7.724      0.000

 Residual Variances
    SISLAMOPH          0.407      0.091      4.487      0.000
```

Islamophobia, zero-inflated negative-binomial model

INPUT INSTRUCTIONS

```
  DATA:        FILE = Danish_majority.txt;
```

[Note: variable list is the same as in the files on OSF and left out here for abbreviation.]

```
  USEVARIABLES =
  Subject
  ISLAMOPH
  WaveD
  CondD;

  WITHIN = WaveD;
  BETWEEN = CondD;
  CLUSTER = Subject;

  COUNT ARE ISLAMOPH (nbi);

  MISSING ARE ALL (-9);
  USEOBSERVATIONS ARE (DoubleChosen EQ 0) AND (GenAgeMat EQ 0);

  ANALYSIS: TYPE = TWOLEVEL RANDOM;

  MODEL:

  %WITHIN%
  sISLAMOPH | ISLAMOPH ON WaveD;

  %BETWEEN%
  ISLAMOPH ON CondD;
  sISLAMOPH ON CondD;

  OUTPUT: SAMPSTAT TECH1;

  PLOT: TYPE = Plot3;
```

SUMMARY OF ANALYSIS

```
Number of groups                                   1
Number of observations                           440
```

COUNT PROPORTION OF ZERO, MINIMUM AND MAXIMUM VALUES

```
     ISLAMOPH    0.505        0       48
```

SAMPLE STATISTICS

SAMPLE STATISTICS

Means

	WAVED	CONDD
1	0.318	0.514

Covariances

	WAVED	CONDD
WAVED	0.217	
CONDD	-0.004	0.250

Correlations

	WAVED	CONDD
WAVED	1.000	
CONDD	-0.019	1.000

THE MODEL ESTIMATION TERMINATED NORMALLY

MODEL FIT INFORMATION

Number of Free Parameters 8

Loglikelihood

H0 Value -936.707
H0 Scaling Correction Factor 0.9114
for MLR

Information Criteria

Akaike (AIC) 1889.415
Bayesian (BIC) 1922.109
Sample-Size Adjusted BIC 1896.721
(n* = (n + 2) / 24)

MODEL RESULTS

	Estimate	S.E.	Est./S.E.	Two-Tailed P-Value
**Within Level**				
Dispersion				
ISLAMOPH	0.291	0.124	2.346	0.019
**Between Level**				
SISLAMOPH ON				
CONDD	-0.920	0.273	-3.363	0.001
ISLAMOPH ON				
CONDD	1.694	0.299	5.674	0.000
Means				
ISLAMOPH#1	-0.596	0.204	-2.920	0.003
Intercepts				
SISLAMOPH	1.224	0.252	4.848	0.000
ISLAMOPH	0.019	0.327	0.057	0.955
Residual Variances				
SISLAMOPH	0.000	0.000	4.044	0.000
ISLAMOPH	1.007	0.251	4.013	0.000

Ethnic persecution (Posse scale), zero-inflated Poisson model

INPUT INSTRUCTIONS

DATA: FILE = Danish_majority.txt;

[Note: variable list is the same as in the files on OSF and left out here for abbreviation.]

```
USEVARIABLES =
Subject
POSS
WaveD
CondD;

WITHIN = WaveD;
BETWEEN = CondD;
CLUSTER = Subject;

COUNT ARE POSS (pi);

MISSING ARE ALL (-9);
USEOBSERVATIONS ARE (DoubleChosen EQ 0) AND (GenAgeMat EQ 0);

ANALYSIS: TYPE = TWOLEVEL RANDOM;

MODEL:

%WITHIN%
sPOSS | POSS ON WaveD;

%BETWEEN%
POSS ON CondD;
sPOSS ON CondD;

OUTPUT: SAMPSTAT TECH1;

SAVEDATA: FILE IS Danish_majority_out.dat;

PLOT: TYPE = Plot3;
```

SUMMARY OF ANALYSIS

Number of groups 1
Number of observations 447

COUNT PROPORTION OF ZERO, MINIMUM AND MAXIMUM VALUES

POSS	0.582	0	36

SAMPLE STATISTICS

SAMPLE STATISTICS

Means

	WAVED	CONDD
1	0.313	0.515

Covariances

	WAVED	CONDD
WAVED	0.215	
CONDD	-0.005	0.250

Correlations

	WAVED	CONDD
WAVED	1.000	
CONDD	-0.020	1.000

THE MODEL ESTIMATION TERMINATED NORMALLY

MODEL FIT INFORMATION

Number of Free Parameters 6

Loglikelihood

H0 Value -1051.231
H0 Scaling Correction Factor 3.8654
for MLR

Information Criteria

Akaike (AIC) 2114.461
Bayesian (BIC) 2139.077
Sample-Size Adjusted BIC 2120.035
(n* = (n + 2) / 24)

MODEL RESULTS

	Estimate	S.E.	Two-Tailed Est./S.E.	P-Value
Within Level				
Between Level				
**SPOSS ON**				
**CONDD**	**-0.697**	**0.396**	**-1.760**	**0.078**
POSS ON				
CONDD	1.058	0.371	2.849	0.004
Means				
POSS#1	0.297	0.104	2.855	0.004
Intercepts				
SPOSS	0.279	0.376	0.742	0.458
POSS	1.269	0.353	3.597	0.000
Residual Variances				
SPOSS	0.349	0.072	4.865	0.000

Ethnic persecution (Posse scale), zero-inflated negative-binomial model

INPUT INSTRUCTIONS

```
DATA:         FILE = Danish_majority.txt;

[Note: variable list is the same as in the files on OSF and left out here for abbreviation.]

  USEVARIABLES =
Subject
POSS
WaveD
CondD
;

WITHIN = WaveD;
BETWEEN = CondD;
CLUSTER = Subject;

COUNT ARE POSS (nbi);

MISSING ARE ALL (-9);
USEOBSERVATIONS ARE (DoubleChosen EQ 0) AND (GenAgeMat EQ 0);

ANALYSIS: TYPE = TWOLEVEL RANDOM;

MODEL:

%WITHIN%
sPOSS | POSS ON WaveD;

%BETWEEN%
POSS ON CondD;
sPOSS ON CondD;

OUTPUT: SAMPSTAT TECH1;

SAVEDATA: FILE IS Danish_majority_out.dat;

PLOT: TYPE = Plot3;
```

SUMMARY OF ANALYSIS

```
Number of groups                                   1
Number of observations                           447
```

COUNT PROPORTION OF ZERO, MINIMUM AND MAXIMUM VALUES

```
    POSS       0.582       0       36
```

SAMPLE STATISTICS

SAMPLE STATISTICS

Means

	WAVED	CONDD
1	0.313	0.515

Covariances

	WAVED	CONDD
WAVED	0.215	
CONDD	-0.005	0.250

Correlations

	WAVED	CONDD
WAVED	1.000	
CONDD	-0.020	1.000

THE MODEL ESTIMATION TERMINATED NORMALLY

MODEL FIT INFORMATION

Number of Free Parameters 8

Loglikelihood

H0 Value -824.875
H0 Scaling Correction Factor 1.0468
for MLR

Information Criteria

Akaike (AIC) 1665.750
Bayesian (BIC) 1698.571
Sample-Size Adjusted BIC 1673.182
(n* = (n + 2) / 24)

MODEL RESULTS

	Estimate	S.E.	Two-Tailed Est./S.E.	P-Value
**Within Level**				
Dispersion				
POSS	0.175	0.104	1.674	0.094
**Between Level**				
**SPOSS ON**				
**CONDD**	**-1.308**	**0.320**	**-4.091**	**0.000**
POSS ON				
CONDD	1.865	0.324	5.753	0.000
Means				
POSS#1	-0.159	0.158	-1.005	0.315
Intercepts				
SPOSS	1.521	0.300	5.070	0.000
POSS	-0.178	0.327	-0.546	0.585
Residual Variances				
SPOSS	0.001	0.000	4.202	0.000
POSS	0.850	0.233	3.649	0.000

Additional Linear Models

For the main analyses, we focused on zero-inflated Poisson regressions because the data was heavily skewed and the modal response was minimal endorsement of violent intentions. Still, since we did not fully anticipate this or pre-register a model that is better suited to handle such a distribution, we also present results below of a simpler, linear model (all other model specifications are the same as in the main results). The predicted effects were significant also in these models. Again, we do not believe our hypothesis should be tested with the models shown below, but we present them here for full transparency. *The key result – the cross-level wave × condition interaction – is highlighted in boldface.*

Linear Model for Non-Muslim Sample

```
INPUT INSTRUCTIONS

  DATA:         FILE = Danish_majority.txt;
```

[Note: variable list is the same as in the files on OSF and left out here for abbreviation.]

```
  USEVARIABLES =
  Subject
  VBI
  WaveD
  CondD;

  WITHIN = WaveD;
  BETWEEN = CondD;
  CLUSTER = Subject;

  MISSING ARE ALL (-9);
  USEOBSERVATIONS ARE (DoubleChosen EQ 0) AND (GenAgeMat EQ 0);

  ANALYSIS: TYPE = TWOLEVEL RANDOM;

  MODEL:

  %WITHIN%
  sVBI | VBI ON WaveD;

  %BETWEEN%
  VBI ON CondD;
  sVBI ON CondD;

  OUTPUT: SAMPSTAT TECH1;

  SAVEDATA: FILE IS Danish_majority_out.dat;

  PLOT: TYPE = Plot3;

SUMMARY OF ANALYSIS
```

Number of groups 1
Number of observations 464

SUMMARY OF DATA

Number of missing data patterns 1
Number of clusters 325

Average cluster size 1.428

Estimated Intraclass Correlations for the Y Variables

Variable	Intraclass Correlation	Variable	Intraclass Correlation
VBI	0.462		

SAMPLE STATISTICS

NOTE: The sample statistics for within and between refer to the maximum-likelihood estimated within and between covariance matrices, respectively.

ESTIMATED SAMPLE STATISTICS FOR WITHIN

Means

	VBI	WAVED	CONDD
1	0.000	0.306	0.000

Covariances

	VBI	WAVED	CONDD
VBI	39.546		
WAVED	0.300	0.212	
CONDD	0.000	0.000	0.000

Correlations

	VBI	WAVED	CONDD
VBI	1.000		
WAVED	0.104	1.000	
CONDD	0.000	0.000	0.000

ESTIMATED SAMPLE STATISTICS FOR BETWEEN

Means

	VBI	WAVED	CONDD
1	10.105	0.000	0.517

Covariances

	VBI	WAVED	CONDD
VBI	33.999		
WAVED	0.000	0.000	
CONDD	1.357	0.000	0.250

Correlations

	VBI	WAVED	CONDD
VBI	1.000		
WAVED	0.000	0.000	
CONDD	0.466	0.000	1.000

MAXIMUM LOG-LIKELIHOOD VALUE FOR THE UNRESTRICTED (H1) MODEL IS -1617.026

THE MODEL ESTIMATION TERMINATED NORMALLY

MODEL FIT INFORMATION

Number of Free Parameters 7

Loglikelihood

H0 Value -1614.806
H0 Scaling Correction Factor 1.5229
for MLR

Information Criteria

Akaike (AIC) 3243.613
Bayesian (BIC) 3272.592
Sample-Size Adjusted BIC 3250.376
(n* = (n + 2) / 24)

MODEL RESULTS

	Estimate	S.E.	Est./S.E.	Two-Tailed P-Value
**Within Level**				
Residual Variances				
VBI	37.153	7.421	5.006	0.000
**Between Level**				
**SVBI ON**				
**CONDD**	**2.857**	**1.376**	**2.076**	**0.038**
VBI ON				
CONDD	4.670	0.896	5.212	0.000
Intercepts				
VBI	7.259	0.616	11.778	0.000
SVBI	-0.018	0.950	-0.019	0.985
Residual Variances				
VBI	27.870	7.392	3.770	0.000
SVBI	1.549	13.299	0.116	0.907

Linear Model for Muslim Sample

INPUT INSTRUCTIONS

```
DATA:       FILE = Muslim_data.txt;
```

[Note: variable list is the same as in the files on OSF and left out here for abbreviation.]

```
USEVARIABLES =
Subject
VBI
WaveD
CondD;

WITHIN = WaveD;
BETWEEN = CondD;
CLUSTER = Subject;

MISSING ARE ALL (-9);
USEOBSERVATIONS ARE (DoubleChosen EQ 0) AND (GenAgeMat EQ 0);

ANALYSIS: TYPE = TWOLEVEL RANDOM;

MODEL:

%WITHIN%
sVBI | VBI ON WaveD;

%BETWEEN%
VBI ON CondD;
sVBI ON CondD;

OUTPUT: SAMPSTAT TECH1;

PLOT: TYPE = Plot3;
```

SUMMARY OF ANALYSIS

```
Number of groups                    1
Number of observations            313
```

SUMMARY OF DATA

```
   Number of missing data patterns         1
   Number of clusters                    214

   Average cluster size        1.463

   Estimated Intraclass Correlations for the Y Variables
```

Variable	Intraclass Correlation	Variable	Intraclass Correlation
VBI	0.739		

SAMPLE STATISTICS

NOTE: The sample statistics for within and between refer to the maximum-likelihood estimated within and between covariance matrices, respectively.

ESTIMATED SAMPLE STATISTICS FOR WITHIN

Means

	VBI	WAVED	CONDD
1	0.000	0.332	0.000

Covariances

	VBI	WAVED	CONDD
VBI	24.508		
WAVED	0.289	0.222	
CONDD	0.000	0.000	0.000

Correlations

	VBI	WAVED	CONDD
VBI	1.000		
WAVED	0.124	1.000	
CONDD	0.000	0.000	0.000

ESTIMATED SAMPLE STATISTICS FOR BETWEEN

Means

	VBI	WAVED	CONDD
1	10.534	0.000	0.589

Covariances

	VBI	WAVED	CONDD

VBI	69.346		
WAVED	0.000	0.000	
CONDD	1.566	0.000	0.242

Correlations

	VBI	WAVED	CONDD
VBI	1.000		
WAVED	0.000	0.000	
CONDD	0.382	0.000	1.000

MAXIMUM LOG-LIKELIHOOD VALUE FOR THE UNRESTRICTED (H1) MODEL IS -1101.582

THE MODEL ESTIMATION TERMINATED NORMALLY

MODEL FIT INFORMATION

Number of Free Parameters 7

Loglikelihood

H0 Value -1093.833
H0 Scaling Correction Factor 1.2658
for MLR

Information Criteria

Akaike (AIC) 2201.667
Bayesian (BIC) 2227.890
Sample-Size Adjusted BIC 2205.688
(n* = (n + 2) / 24)

MODEL RESULTS

	Estimate	S.E.	Est./S.E.	Two-Tailed P-Value
**Within Level**				
Residual Variances				
VBI	17.343	6.153	2.819	0.005

Between Level

**SVBI ON**				
**CONDD**	**5.122**	**1.211**	**4.228**	**0.000**
VBI ON				
CONDD	5.057	1.277	3.959	0.000
Intercepts				
VBI	7.156	1.000	7.154	0.000
SVBI	-1.446	0.913	-1.585	0.113
Residual Variances				
VBI	64.383	8.468	7.603	0.000
SVBI	5.693	11.694	0.487	0.626
